# A noncanonical meiotic cohesin complex associates with specific MCM complexes to promote cohesion establishment and homolog bias

**DOI:** 10.1126/sciadv.aej1008

**Published:** 2026-10-23

**Authors:** Lujie Ma, Qianqian Jin, Dongcheng Hu, Xianwu Lin, Zizhen Fan, Rihui Yan

**Affiliations:** ^1^ School of Life and Health Sciences, Hainan University, Haikou, China.; ^2^ School of Tropical Agriculture and Forestry, Hainan University, Haikou, China.; ^3^ National Nanfan Research Institute, Chinese Academy of Agricultural Sciences, Sanya, China.

## Abstract

A meiosis-specific cohesin complex is essential for the segregation of homologous chromosomes and sister chromatids during meiosis, yet its composition and mechanism in *Drosophila* remain elusive. Here, we demonstrate that SOLO directly interacts with SUNN, SSEP, and SMC1 and that these interactions are conserved between *Drosophila melanogaster* and *Zeugodacus cucurbitae*, a Tephritidae species within Diptera. SOLO associates with SMC1 via specific conserved residues within its highly conserved carboxyl-terminal domain. Furthermore, SOLO directly interacts with the MCM4 and MCM6 subunits of the MCM complex and with MMS22L to establish cohesion; consequently, postreplicative expression of SOLO results in a complete function loss. SOLO also interacts with the MEI-217 and MEI-218 subunits of the meiosis-specific minichromosome maintenance complex to promote homolog bias during meiosis. We propose that in Diptera, an evolutionarily diverged meiotic cohesin complex, composed of SOLO, SUNN, SSEP, SMC1, and SMC3, interacts with various complexes to fulfill its diverse meiotic functions.

## INTRODUCTION

Cohesion between sister chromatids is essential for meiosis, during which homologous chromosomes (homologs) segregate during the first division (meiosis I) and sister chromatids segregate during the second division (meiosis II). In most eukaryotes, homologous chromosomes pair and undergo recombination during meiosis I to generate crossovers, ultimately forming chiasmata that are stabilized by sister chromatid cohesion distal to crossover sites (*1*, *2*). Chiasmata are resolved at anaphase I to allow homolog segregation, but sister chromatids remain tethered by centromeric cohesion until anaphase II when cohesion is cleaved to permit sister chromatid segregation (*3*–*5*).

Cohesion is provided by a multiprotein, ring-shaped complex called cohesin. The mitotic cohesin complex consists of four subunits: two structural maintenance of chromosomes (SMC) subunits, SMC1 and SMC3, and two non-SMC subunits, the α-kleisin family member SCC1/RAD21 and SCC3/SA. In most eukaryotes, one or more meiosis-specific subunits replace their mitotic counterparts to form meiotic cohesin complexes. In most of these complexes, the meiosis-specific subunit REC8 replaces the mitotic α-kleisin SCC1/RAD21 (*3*, *6*, *7*). Studies in *Saccharomyces cerevisiae*, *Caenorhabditis elegans*, and other organisms have demonstrated that REC8 is structurally and functionally conserved and plays crucial roles in meiosis. It is essential for homologous recombination, stable chiasma formation, and the proper segregation of homologs during meiosis I and sister chromatids during meiosis II. In *rec8* mutants, SCC1/RAD21 cannot fulfill the meiotic functions of REC8 nor can it protect the cohesin complex from separase cleavage during meiosis I (*8*–*13*).

However, the ortholog of REC8 in *Drosophila melanogaster* has not yet been identified nor has its counterparts in other insects. C(2)M, a distantly related meiosis-specific α-kleisin protein, does not play a role in sister chromatid cohesion but is instead involved in meiotic homologous recombination and synaptonemal complex (SC) formation (*14*–*16*). RAD21, a mitotic α-kleisin protein, is not involved in meiotic cohesion (*17*). Therefore, both C(2)M and RAD21 cannot substitute for REC8 in *Drosophila* meiosis.

Four meiosis-specific genes required for meiotic sister chromatid cohesion have been identified in *Drosophila*: *ord* (*orientation disrupter*), *solo* (*sisters on the loose*), *sunn* (*sisters unbound*), and *ssep* (*sisters separate*). SOLO, SUNN, and ORD are required for centromeric cohesion and SMC1 localization at centromeres during both male and female meiosis in *Drosophila*, as well as for synapsis and recombination in female meiosis (*18*–*22*). In addition, proper localization of SOLO and SUNN at centromeres depends on each other (*21*). Furthermore, SOLO physically interacts with SMC1 in vivo (*19*). Considering the localization differences of SOLO, SUNN, and ORD at early prophase I (*18*, *20*, *21*, *23*), these findings imply the existence of a meiosis-specific cohesin complex containing SMC1, SOLO, and SUNN in *Drosophila*, which is supported by a study conducted by McKim and colleagues (*16*). Recently, SSEP was found to be essential for centromeric cohesion in *Drosophila* meiosis and exhibits phenotypes and localization patterns similar to those of SOLO and SUNN (*23*). Collectively, these studies indicate the existence of a cohesin complex containing SMC1, SOLO, SUNN, and SSEP. However, its composition requires further confirmation, and the interactions among its subunits remain to be elucidated.

Cohesion between sister chromatids is established during the S phase and is coupled to DNA replication (*24*–*26*). In eukaryotic cells, DNA replication initiates at multiple origins during the G_1_ phase through the assembly of pre-replicative complexes (pre-RCs), which contain two hexamers of minichromosome maintenance proteins 2 to 7 (MCM2-MCM7) (*27*, *28*). In vertebrates, the MCM2-MCM7 complex physically interacts with cohesin (*29*, *30*). In *Drosophila*, chromatin immunoprecipitation sequencing has revealed extensive genome-wide overlap between the origin recognition complex binding sites and cohesin loading (*31*), suggesting that the pre-RC is likely required for cohesin loading in *Drosophila*. However, whether the pre-RC is involved in cohesin loading and establishment in *Drosophila* remains elusive. It is also unknown whether similar physical interactions exist between MCM2-MCM7 and cohesin and whether the mechanisms of meiotic cohesin loading and establishment are analogous to those of mitotic cohesin.

Meiotic recombination generates crossovers between homologs through repair of a selected subset of programmed DNA double-strand breaks and is essential for homologous chromosome segregation (*1*, *2*). Meiotic cohesin complexes are required for meiotic crossover formation and recombination (*1*–*3*). In *Drosophila* females (males are achiasmate), recombination and crossover distribution are markedly affected by mutations in SOLO and ORD (*18*, *19*). SOLO and ORD play a role in loading the centromeric SC (*18*, *19*, *32*), which may contribute to meiotic recombination. Meiotic crossovers are also promoted by a meiosis-specific minichromosome maintenance (mei-MCM) complex comprising MCM and MCM-like proteins REC/MCM8, MEI-217, MEI-218, and probably, MCM5 (*33*–*35*). Mutations in *rec*, *mei-217*, *mei-218*, and *Mcm5* lead to severely reduced meiotic crossovers (*36*–*39*). The specific role of the *Drosophila* meiotic cohesin complex in crossover formation remains elusive, as does the precise mechanism of the mei-MCM complex in meiotic recombination.

At anaphase I, the protease separase is activated to remove arm cohesion, thereby releasing chiasmata and allowing homologous chromosomes to segregate. During meiosis I, centromeric cohesion is protected by the conserved protein Shugoshin (MEI-S332 in *Drosophila*), which prevents separase from cleaving cohesin at centromeric regions. At anaphase II, separase is activated again. At this stage, Shugoshin no longer protects centromeric cohesion, allowing separase to remove centromeric cohesion and enabling sister chromatid segregation (*6*, *40*). Two studies have demonstrated that separase is required for proper segregation of homologs during meiosis I and sister chromatids during meiosis II in both male and female *Drosophila* meiosis (*41*, *42*), suggesting its role in cleaving meiotic cohesin. Separase has been found to cleave the α-kleisin Rad21 during mitosis (*8*, *43*–*45*) and the homolog conjunction protein UNO during *Drosophila* male meiosis (*46*). However, mutation of a potential separase cleavage site in SSEP produced only minor effects on chromosome disjunction. Thus, the meiosis-specific cohesin target of separase in *Drosophila* remains to be identified.

To gain insight into the composition and mechanism of the meiotic cohesin complex in *Drosophila*, we investigated the interactions of SOLO with meiotic cohesion proteins and subunits from other complexes. Our results reveal that the N-terminal, middle, and C-terminal regions of SOLO are required for direct interactions with SOLO itself, SUNN, and SMC1, respectively, while SSEP can interact with the full-length SOLO. Our findings also demonstrate that SOLO directly interacts with MCM4 and MCM6 within the MCM complex and with MMS22L and that the establishment of meiotic cohesion during DNA replication is required for SOLO function. In addition, SOLO directly interacts with the MEI-217 and MEI-218 subunits of the mei-MCM complex. Notably, the meiosis-specific proteins SOLO, SUNN, and SSEP are present in Diptera species and exhibit conserved subunit interactions in *Zeugodacus cucurbitae*. Thus, our findings suggest that an evolutionarily diverged, meiosis-specific cohesin complex, composed of SOLO, SUNN, SSEP, SMC1, and SMC3, coordinates with various complexes to execute its diverse meiotic functions.

## RESULTS

### SOLO directly interacts with SUNN, SMC1, SSEP, and itself

A previous reciprocal coimmunoprecipitation (Co-IP) study of SOLO and SMC1 demonstrated that SOLO interacts with SMC1 in ovarian protein extracts from *Drosophila* females (*19*). Moreover, SUNN and SOLO are mutually required for their centromeric localization, and ORD is necessary for the proper positioning of SOLO and SUNN at centromeres (*20*, *21*). SUNN and ORD are also important for the centromeric localization of SSEP (*23*). Given that localization of ORD is extrachromosomal during early G_2_ and on chromosomes by late G_2_ (*18*), these cellular and genetic data suggest that SUNN, SOLO, SSEP, and SMC1 are likely components of the meiotic cohesin complex and that ORD may play a role in maintaining this complex (*16*, *19*–*21*, *23*). However, the interaction nature of these meiotic proteins remains unknown.

To elucidate the interactions among these meiosis-specific proteins, we performed yeast two-hybrid assays. Consistent with previous studies (*19*, *20*), we confirmed a direct interaction of SOLO with SMC1 (Fig. 1A). Furthermore, we identified a robust and stable interaction between SOLO and SUNN, as well as SOLO self-interaction (Fig. 1B and fig. S1A). However, SOLO did not show direct interactions with the cohesion proteins ORD, MEI-S332, C(2)M, and SA1 or with the homologous chromosome conjunction proteins UNO and MNM (Fig. 1B). Neither SMC1 nor SMC3 was found to directly interact with ORD, MEI-S332, C(2)M, SA1, UNO, MNM, or SUNN (Fig. 1B). Moreover, we detected no interactions among ORD, MEI-S332, C(2)M, SA1, or SUNN, except that MEI-S332 interacted with SA1 and itself (fig. S1B).

**Fig. 1.**
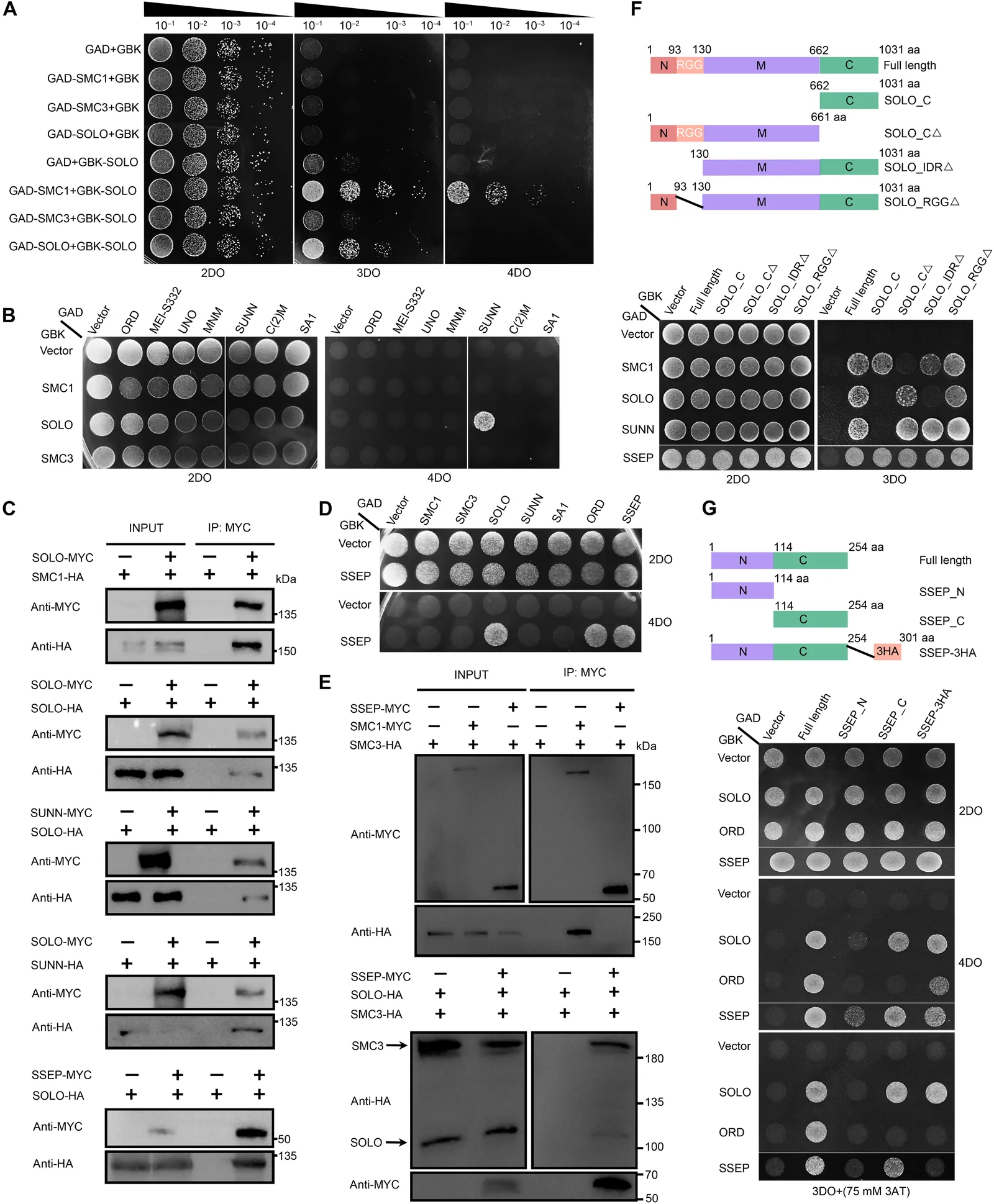
Interactions between SOLO and meiotic proteins. (**A** and **B**) SOLO interacts with SMC1, SUNN, and itself in yeast two-hybrid assays. Yeast cells transformed with the indicated plasmids were grown at 30°C on control 2DO (SD/-Leu, -Trp) and high-stringency 3DO (SD/-Leu, -Trp, -His) and 4DO (SD/-Leu, -Trp, -Ade, -His) plates. (**C**) Co-IP of SOLO with SMC1, SUNN, itself, and SSEP. S2R^+^ cells were cotransfected with the indicated constructs, and IPs were performed using anti–c-Myc or anti-HA antibodies, followed by immunoblotting. (**D**) SSEP interacts with SOLO, ORD, and itself in yeast two-hybrid assays. Yeast cells transformed with the indicated plasmids were grown at 30°C on control 2DO (SD/-Leu, -Trp) and high-stringency 4DO (SD/-Leu, -Trp, -Ade, -His) plates. (**E**) Co-IP of SSEP with SOLO and SMC3. SSEP interacts with SOLO and SMC3, but its interaction with SMC3 was abolished in the absence of SOLO. (**F**) SOLO domains interacting with SMC1, SUNN, SSEP, and itself in yeast two-hybrid assays. Yeast cells transformed with the indicated plasmids were grown at 30°C on control 2DO (SD/-Leu, -Trp) and high-stringency 3DO (SD/-Leu, -Trp, -His) plates. (**G**) SSEP domains interacting with SOLO and ORD in yeast two-hybrid assays. Yeast cells transformed with the indicated plasmids were grown at 30°C on control 2DO (SD/-Leu, -Trp), high-stringency 4DO (SD/-Leu, -Trp, -Ade, -His) plates, and 3DO plates supplemented with 75 mM 3-aminotriazole (3AT).

To confirm whether these interactions identified by yeast two-hybrid analyses also occur in vivo, we conducted Co-IP assays in S2R^+^ cells. The results demonstrated that SOLO interacts with SMC1, SUNN, and itself, which is consistent with the yeast two-hybrid data (Fig. 1C). In agreement with predictions from AlphaFold3 (*23*), SSEP exhibited strong interactions with SOLO, ORD, and itself (Fig. 1D and fig. S1C). Together, these results demonstrate direct interactions of SOLO with SMC1, SUNN, and SSEP, with SOLO and SSEP each capable of self-association. Although direct interaction between SSEP and SMC3 was not detected in yeast two-hybrid assays, SMC3 was coimmunoprecipitated by SSEP when S2R^+^ cells were cotransfected with both SOLO and SSEP but not with SMC1 and SSEP (Fig. 1E), suggesting that SMC3, SSEP, and SOLO are within the same complex.

We further examined the interactions of different SOLO domains with SMC1, SUNN, SSEP, and SOLO itself. Consistent with previous investigations of three *solo* mutants (*19*, *20*), the C-terminal region (residues 662 to 1031) of SOLO is required for its interaction with SMC1 (Fig. 1F). Amino acid residues 130 to 661 mediate the interaction with SUNN (Fig. 1F), while residues 1 to 93 are required for SOLO self-interaction. Multiple regions of SOLO interact with SSEP (Fig. 1F). Deletion of the RGG domain does not affect the interaction of SOLO with SMC1, SUNN, SSEP, or SOLO itself (Fig. 1F). We also examined the region of SSEP responsible for interacting with SOLO, ORD, and SSEP itself. The C terminus of SSEP exhibited a strong interaction with SOLO and SSEP itself (Fig. 1G), whereas its N terminus did not. In contrast, the interaction of ORD with SSEP requires full-length SSEP, as neither the N terminus nor C terminus alone interacted with SSEP. Furthermore, adding a 3HA tag to the C terminus of full-length SSEP weakened its interaction with ORD and SSEP itself but did not affect its interaction with SOLO (Fig. 1G). These results may explain the functional defect observed in the *ssep^Z2-4635^* mutant (*23*).

To test the effects of SOLO self-interaction on cohesin complex stability and sister chromatid segregation, we generated transgenic *Drosophila* lines expressing SOLO lacking either residues 1 to 93 or the RGG domain and evaluated their effects on chromosome segregation. Loss of SOLO self-interaction resulted in only 3.93% nondisjunction (NDJ) (table S1), suggesting a minimal impact on chromosome segregation and therefore indicating little effect on the stability of the cohesin complex. This finding aligns with a study in human cells in which disruption of RAD21 self-interaction does not affect cohesin complex stability (*47*). In contrast, deletion of the RGG domain led to a considerably higher NDJ frequency of 42.2% (table S1). However, NDJ frequencies associated with the C-terminal *solo* mutants consistently remained around 50% (*19*, *20*). This suggests that the meiotic defects caused by RGG domain deletion may not result from disruption of the interaction between SOLO and SMC3. In eukaryotes, the interaction interfaces between SMC1/SMC3 and kleisin proteins are evolutionarily conserved (*48*). However, the N-terminal region of SOLO lacks any clearly conserved motifs for such binding. Therefore, the shared presence of the RGG domain in both SOLO and VASA (VAS) may be related to nonstructural roles of SOLO, such as RNA/DNA binding (*49*).

### Conservation of SOLO, SUNN, SSEP, ORD, and their interactions

A previous study revealed a unique gene structure shared by *solo* and *vas*, in which *solo* shares its first three exons with *vas*, while its last two exons are located within the third large intron of *vas* (*20*). This arrangement generates two distinct transcripts via alternative splicing: the *solo* transcript that comprises the three shared upstream exons and the two *solo*-specific intronic exons, creating a continuous open reading frame that encodes a protein of 1031 amino acids in length, and the *vas* transcript that includes the three shared exons and the five *vas*-specific downstream exons, which encode the RNA helicase domain. We investigated the conservation of this gene structure in insects and found its predominant presence in Diptera, with the highest prevalence in the infraorder Brachycera. In most cases, the predicted *solo* genes contain three to four upstream exons shared with *vas* and two *solo*-specific downstream exons embedded within a large intron of *vas*, similar to the gene organization in *Drosophila* (fig. S2), suggesting a conserved gene structure and alternative splicing pattern of *solo* and *vas*. In addition, sequence alignments of predicted SOLO proteins from dipteran species revealed a high degree of C-terminal conservation across families, including Drosophilidae, Tephritidae, and Muscidae (fig. S3). Overall, SUNN is more conserved than SOLO (fig. S4). SSEP and ORD are also present in a variety of dipteran insects (figs. S5 and S6). We cloned the predicted *smc1* (gene ID: 105215847; *Zcsmc1*), *solo* (gene ID: 105217981; *Zcsolo*), and *sunn* (gene ID: 105208535; *Zcsunn*) genes from *Z. cucurbitae* and validated their interactions through yeast two-hybrid and Co-IP assays. As expected, ZcSOLO directly interacted with ZcSMC1, ZcSUNN, and itself (Fig. 2, A and B). We also cloned *Zcssep* (gene ID: 105214577) and *Zcord* (gene ID: 105208593) and found that ZcSSEP directly interacts with ZcSOLO, ZcORD, and itself in yeast two-hybrid assays (Fig. 2C). Furthermore, interaction analyses of truncated variants of ZcSOLO with other meiotic cohesin subunits revealed similar interaction patterns to those observed in *Drosophila*: ZcSOLO interacts with ZcSMC1, ZcSUNN, ZcSSEP, and itself through its C terminus, middle region, multiple regions, and N terminus, respectively (Fig. 2D). Together, these results clearly demonstrate conserved direct interaction patterns of these cohesin subunits between *D. melanogaster* and *Z. cucurbitae*, indicating that the interactions of meiotic cohesin subunits are evolutionarily conserved across dipteran insects despite sequence divergence in some individual components.

**Fig. 2.**
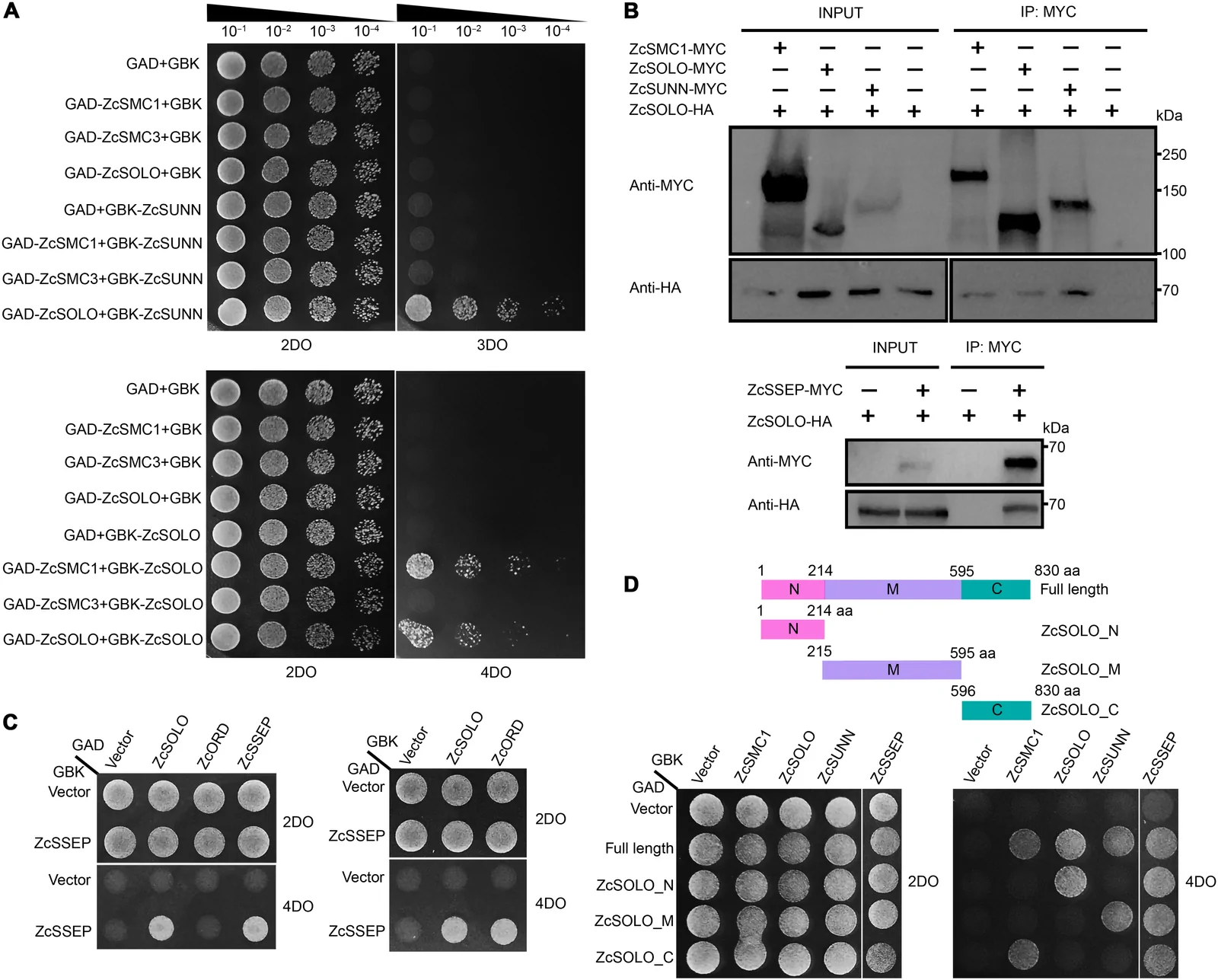
Interactions of ZcSOLO with ZcSUNN, ZcSMC1, ZcSSEP, and itself. (**A**) ZcSOLO interacts with ZcSMC1, ZcSUNN, and itself in yeast two-hybrid assays. Yeast cells transformed with the indicated plasmids were grown at 30°C on control 2DO (SD/-Leu, -Trp), 3DO (SD/-Leu, -Trp, -His), and 4DO (SD/-Leu, -Trp, -Ade, -His) plates. (**B**) ZcSOLO interacts with ZcSMC1, ZcSUNN, ZcSSEP, and itself in Co-IP assays. S2R^+^ cells were cotransfected with pUAST-ZcSOLO-3HA and pUAST-ZcSMC1/ZcSUNN/ZcSOLO-13MYC and cultured at 25°C for 48 hours before analysis. (**C**) ZcSSEP interacts with ZcSOLO, ZcORD, and itself in yeast two-hybrid assays. Yeast cells transformed with the indicated plasmids were grown at 30°C on control 2DO (SD/-Leu, -Trp) and high-stringency 4DO (SD/-Leu, -Trp, -Ade, -His) plates. (**D**) ZcSOLO domains interacting with ZcSMC1, ZcSUNN, ZcSSEP, and itself in yeast two-hybrid assays.

### The conserved C-terminal domain of SOLO interacts with SMC1

Given the sequence similarity of the SOLO C terminus between *D. melanogaster* and *Z. cucurbitae* (Fig. 3A) and the interactions between the SOLO C terminus and SMC1 (Figs. 1F and 2D), we speculated that the interaction between SOLO and SMC1 is conserved between these two species. We found that ZcSOLO directly interacted with DmSMC1 in yeast two-hybrid experiments (Fig. 3B). This interaction was confirmed by Co-IP, as DmSMC1-HA was pulled down by an anti-MYC antibody from extracts of S2R^+^ cells cotransfected with DmSMC1-HA and ZcSOLO-MYC (Fig. 3C).

**Fig. 3.**
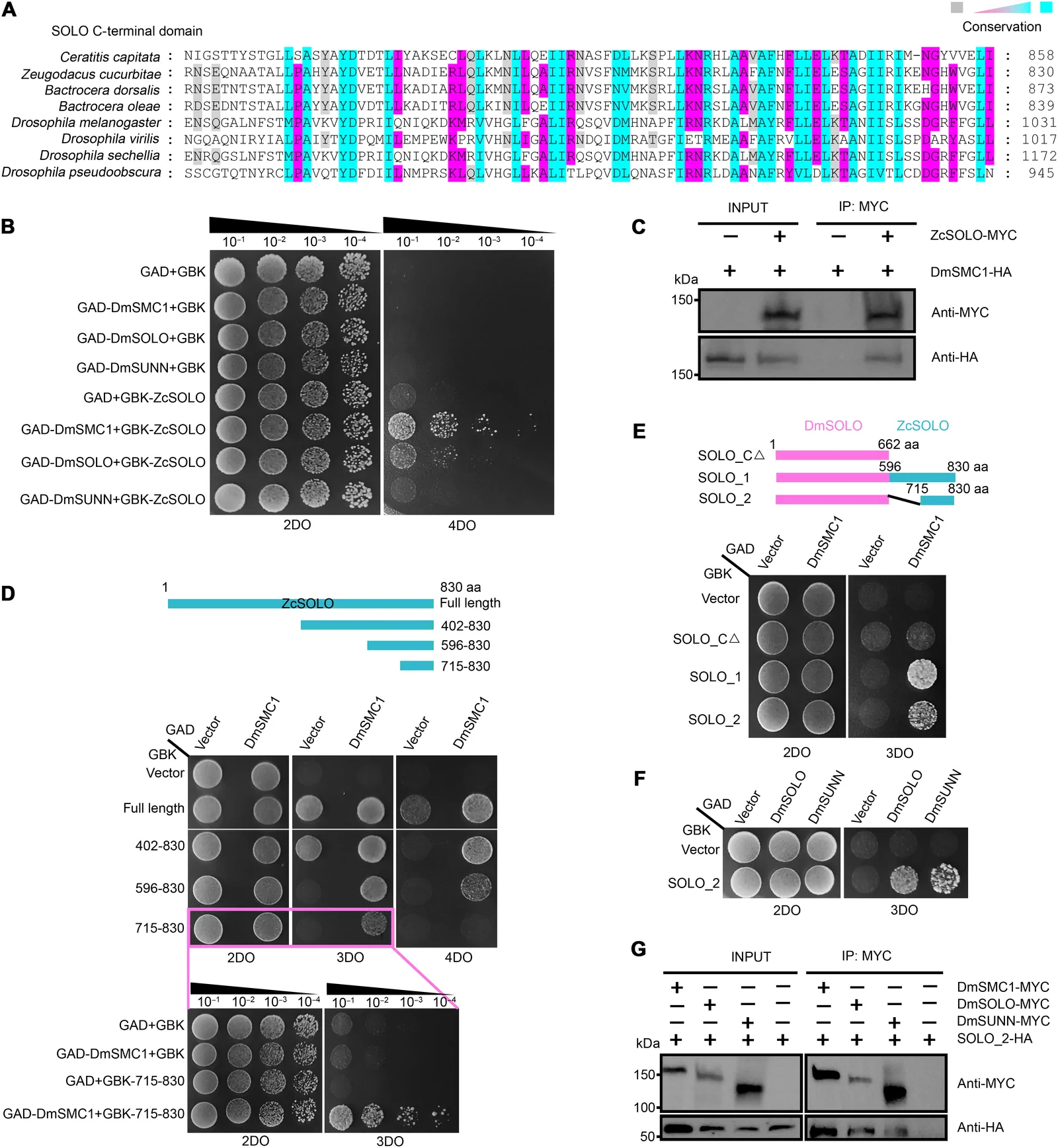
ZcSOLO interacts with DmSMC1 through its conserved C terminus. (**A**) Sequence alignment of SOLO C termini in Drosophilidae and Tephritidae. Amino acid conservation is indicated by increasing color intensity from white to gray, pink, and blue. The protein sequences used for alignment were obtained from fig. S3. (**B**) ZcSOLO interacts with DmSMC1 in yeast two-hybrid assays. Yeast cells transformed with the indicated plasmids were grown at 30°C on control 2DO (SD/-Leu, -Trp) and high-stringency 4DO (SD/-Leu, -Trp, -Ade, -His) plates. (**C**) Co-IP of ZcSOLO with DmSMC1. S2R^+^ cells were cotransfected with pUAST-ZcSOLO-13MYC and pUAST-DmSMC1-3HA and cultured at 25°C for 48 hours before the assay. (**D**) Yeast two-hybrid assay mapping the minimal C-terminal domain of ZcSOLO required for interaction with DmSMC1. (**E**) Yeast two-hybrid assays for interaction of ZcSOLO fusion proteins with DmSMC1. DmSOLO residues 1 to 662 were fused to ZcSOLO residues 596 to 830 (SOLO_1) or 715 to 830 (SOLO_2). (**F**) The SOLO_2 fusion protein interacts with DmSOLO and DmSUNN in yeast two-hybrid assays. (**G**) Co-IP of the SOLO_2 fusion protein with DmSMC1, DmSOLO, and DmSUNN.

SMC1 proteins are highly conserved in sequence and structure (*50*), raising the question of whether the C-terminal region of ZcSOLO can interact with DmSMC1. Using yeast two-hybrid assays with truncated ZcSOLO variants, we determined that the shortest fragment capable of interacting with DmSMC1 is the C-terminal region spanning residues 715 to 830 (Fig. 3D). If residues 715 to 830 are critical for the interaction between SOLO and SMC1 in both species, then these residues may be able to compensate for the functional defects caused by C-terminal deletion of SOLO. To test whether the C terminus of DmSOLO can be functionally replaced by the C terminus of ZcSOLO, we generated fusion proteins combining the truncated DmSOLO (lacking its C terminus, DmSOLO_CΔ) with ZcSOLO residues 596 to 830 (SOLO_1) or 715 to 830 (SOLO_2). Both fusion proteins interacted with DmSMC1 (Fig. 3E). Moreover, the chimeric SOLO protein in which DmSOLO was fused with residues 715 to 830 of the ZcSOLO C terminus still interacted with DmSMC1, DmSUNN, and DmSOLO in yeast two-hybrid and Co-IP experiments (Fig. 3, F and G).

The finding that the chimeric SOLO protein can interact with DmSMC1 in vivo suggests that it may functionally replace endogenous SOLO in *Drosophila* meiosis. To test this possibility, we generated transgenic flies expressing either full-length wild-type ZcSOLO or the chimeric SOLO protein (SOLO_2), in which DmSOLO residues 1 to 662 were fused to ZcSOLO residues 715 to 830. We then conducted genetic crosses to determine whether these transgenes could rescue the NDJ phenotype caused by the *solo* mutation. Full-length ZcSOLO failed to rescue the *solo* mutant phenotype; however, the chimeric SOLO_2 partially rescued the high NDJ frequency observed in *solo* mutants (table S2). This partial rescue may result from structural alterations introduced by the fusion, which may prevent complete restoration of SOLO function. Alternatively, species-specific differences outside the C-terminal region may also contribute to this incomplete functional complementation.

Given that SOLO, like most α-kleisins, interacts with SMC1 through its C-terminal region, we further investigated the details of SOLO-SMC1 interaction. We first aligned the C-terminal domain of SOLO from dipteran species and identified a highly conserved region with identical amino acid residues across species (Fig. 4A). We next aligned DmSOLO with REC8 proteins from *S. cerevisiae*, *Schizosaccharomyces pombe*, *C. elegans*, *Mus musculus*, and *Homo sapiens*, as well as the *Drosophila* C(2)M protein, which is considered as a distant homolog of REC8 but does not function in sister chromatid cohesion (*15*), and identified six highly conserved amino acid residues (Fig. 4B). Given that the C-terminal region of α-kleisins is typically involved in SMC1 interaction, these conserved residues may play a direct role in this interaction. We mutated these residues and performed Co-IP and yeast two-hybrid assays to investigate whether these mutations affect the interaction between SOLO and SMC1 in *D. melanogaster* and *Z. cucurbitae*. The results revealed that DmSOLO mutations such as Leu^1031^→Ser (L1013S), Glu^1012^→Lys (E1012K), Leu^1011^→Ser (L1011S), Leu^1010^→Ser (L1010S), and Arg^1000^→Glu (R1000E) resulted in a complete loss of interaction with SMC1 (Fig. 4, C and D). Likewise, ZcSOLO mutations such as Leu^812^→Ser (L812S), Glu^811^→Lys (E811K), and Arg^799^→Glu (R799E) led to a complete loss of interaction with SMC1 (Fig. 4, C and D). These results indicate that the conserved interaction between the C-terminal domain of α-kleisin proteins and SMC1 depends on these key residues.

**Fig. 4.**
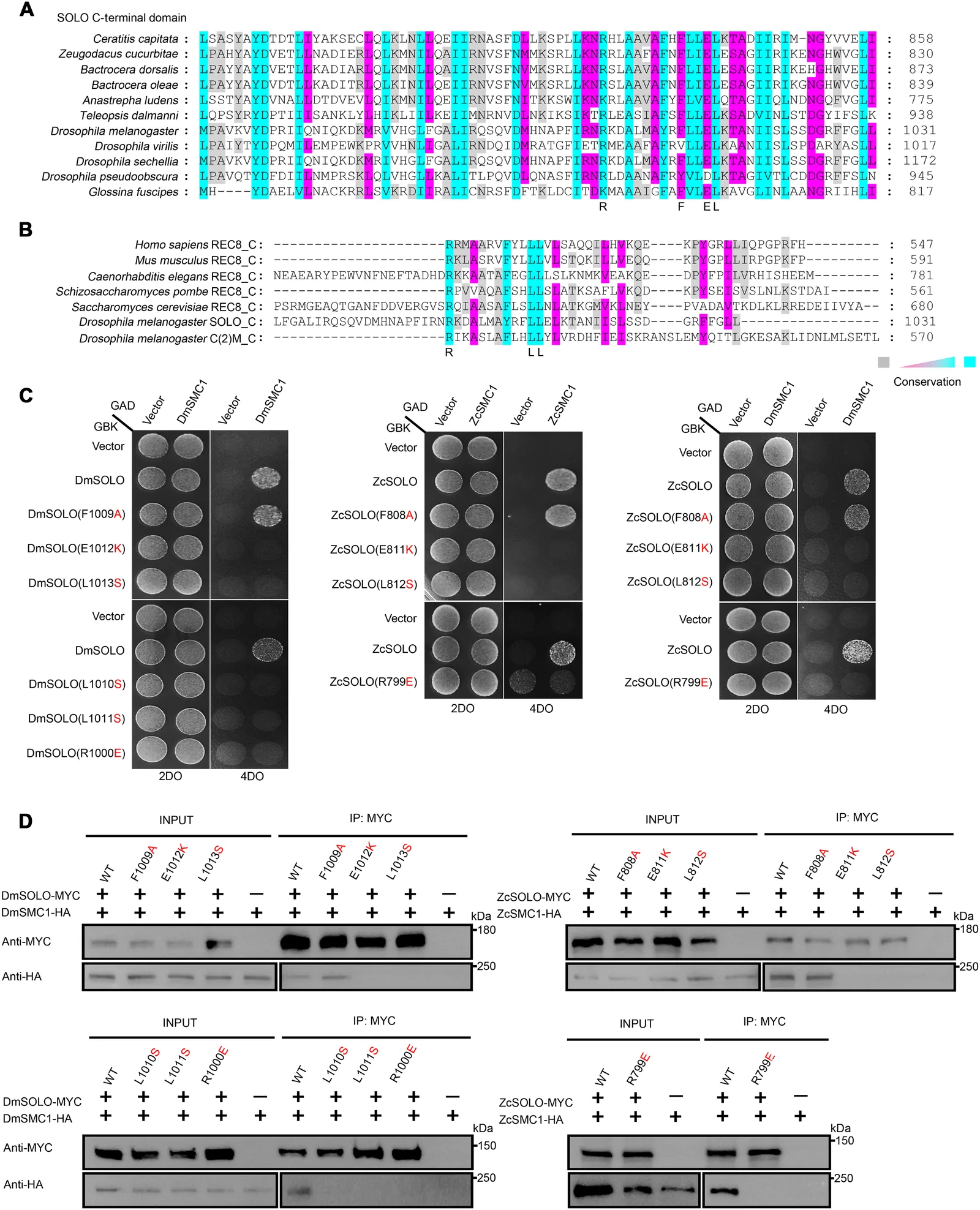
Identification of conserved amino acid residues required for the SOLO-SMC1 interaction. (**A** and **B**) Sequence alignment of SOLO C termini from Diptera with REC8 proteins from *S. cerevisiae*, *C. elegans*, *M. musculus* and *H. sapiens*, as well as with the C(2)M protein from *D. melanogaster*. Amino acid conservation is indicated by increasing color intensity from white to gray, pink, and blue. The protein sequences used for alignment were obtained from GenBank under the following accession numbers: *Ceratitis capitata* (XP_012154950.1), *Z. cucurbitae* (XP_054083496.1), *Bactrocera dorsalis* (XP_049318406.1), *Bactrocera oleae* (XP_036229847.2), *Anastrepha ludens* (XP_053960823.1), *Teleopsis dalmanni* (XP_037957871.1), *D. melanogaster* (NP_001027274.2), *Drosophila virilis* (XP_015028993.1), *Drosophila sechellia* (XP_032573259.1), *Drosophila pseudoobscura* (XP_033235830.1), *Glossina fuscipes* (XP_037882660.1), *H. sapiens* (NP_001041670.1), *M. musculus* (NP_064386.2), *C. elegans* (NP_001255727.1), *S. pombe* (NP_596059.1), *S. cerevisiae* (NP_015332.1), and *D. melanogaster* C(2)M (NP_609788.1). (**C** and **D**) Yeast two-hybrid (C) and Co-IP (D) assays assessing the effects of mutations in conserved C-terminal residues of *D. melanogaster* and *Z. cucurbitae* SOLO on its interaction with SMC1. WT, wild type.

### SUNN interacts with SOLO through its conserved SA domain

Bioinformatics analysis revealed that SUNN shares structural features characteristic of SA proteins, leading to its broad acceptance as the SA subunit of the meiotic cohesin complex in *Drosophila* (*21*). In yeast, mutations of conserved amino acid residues in the conserved and essential surface (CES) region of SA/SCC3 impair its function (*51*). Through multiple sequence alignment of SUNN with SA proteins from model organisms, we identified conserved regions in SUNN that are highly homologous to the CES of SCC3 (fig. S7A). We therefore hypothesized that if SUNN functions as the SA subunit, mutations in these conserved residues might affect its interaction with SOLO. We first tested the region spanning residues 252 to 325 in DmSUNN, which shows the highest conservation with the CES of SCC3. Yeast two-hybrid and Co-IP assays showed that deletion of this region impairs the interaction of DmSUNN with DmSOLO (fig. S7, B and C). Moreover, single amino acid substitutions within this region, including Tyr^252^→Ala (Y252A), Arg^260^→Ala (R260A), Asp^294^→Ala (D294A), Leu^308^→Ala (L308A), Phe^325^→Ala (F325A), and Val^343^→Lys (V343K), disrupted the interaction of DmSUNN with DmSOLO (fig. S7, B and C). We next asked whether mutating the conserved residues in ZcSUNN similarly affects its interaction with ZcSOLO. As expected, the Leu^292^→Ala (L292A) and Phe^309^→Ala (F309A) mutations both disrupted the interaction of ZcSUNN with ZcSOLO (fig. S7, D and E). Collectively, these results demonstrate that SUNN interacts with SOLO through a conserved SA domain in both *D. melanogaster* and *Z. cucurbitae*.

### Loading of SOLO-containing meiotic cohesin complex is coupled to DNA replication

To further explore the mechanism of SOLO in meiosis, we performed a proteomic screen to identify proteins that interact with SOLO. Co-IP and mass spectrometry were carried out using ovary extracts from *Drosophila* females expressing FLAG::SOLO under the control of *nanos*-GAL4::VP16, and the proteins interacting with SOLO were identified (table S3). Across four independent experiments, SOLO was consistently enriched, confirming the reliability of the immunoprecipitation (IP). Unexpectedly, only a few cohesin proteins were enriched despite the results showing that SOLO is a core subunit of the cohesin complex. The core components SMC1, SMC3, and SUNN, as well as Nipped-B, were only weakly enriched. Components of the MCM complex were highly enriched in all four Co-IP experiments, and the intact MCM2-MCM7 complex was identified by Co-IP and mass spectrometry (table S3). To determine whether the enrichment of the MCM complex by SOLO reflects direct interactions, we investigated the interactions between SOLO and each of the seven MCM subunits using the yeast two-hybrid system. The results demonstrated that DmSOLO directly interacts with DmMCM4 and DmMCM6 (Fig. 5A), and these interactions were verified by Co-IP (Fig. 5B). MMS22L, which functions together with the MCM complex in cohesion establishment (*52*–*54*), was also found to directly interact with SOLO (Fig. 5A and table S3). In addition, we identified several DNA repair–related proteins. However, they did not show direct interactions with SOLO (Fig. 5C), suggesting that their association may be indirect, probably mediated by DNA strand interactions or through indirect associations of SOLO with DNA repair complexes.

**Fig. 5.**
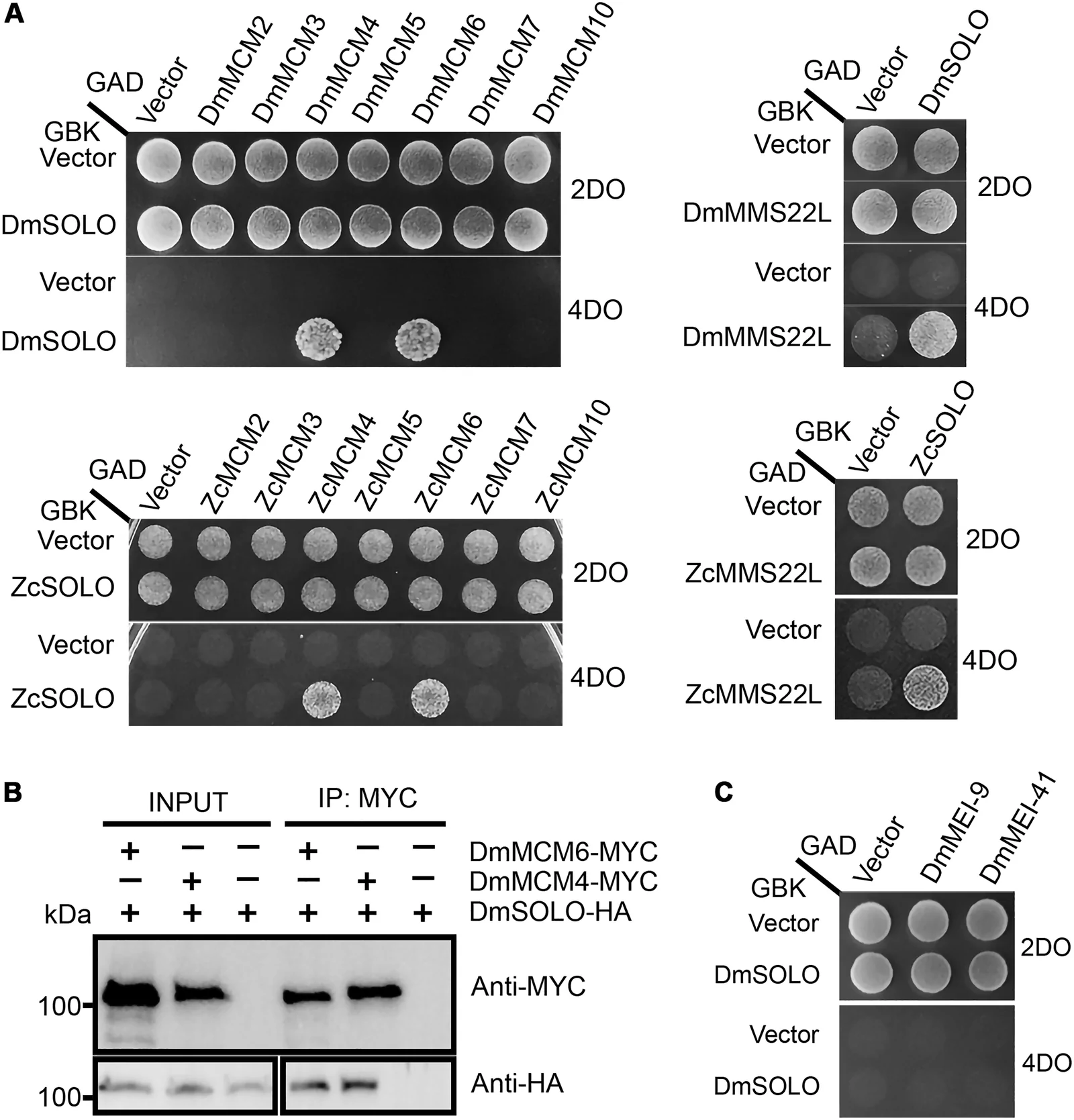
Interaction of SOLO and MCM complex components. (**A**) Yeast two-hybrid assays show that SOLO interacts with the MCM4 and MCM6 subunits in the MCM complex, as well as MMS22L, in both *D. melanogaster* and *Z. cucurbitae*. Yeast cells transformed with the indicated plasmids were grown at 30°C on control 2DO (SD/-Leu, -Trp) and high-stringency 4DO (SD/-Leu, -Trp, -Ade, -His) plates. (**B**) Co-IP of DmSOLO with DmMCM4 and DmMCM6. S2R^+^ cells were cotransfected with pUAST-DmSOLO-3HA and pUAST-DmMCM4/6-13MYC and cultured at 25°C for 48 hours before the assay. (**C**) DmSOLO does not directly interact with DmMEI-9 or DmMEI-41 in yeast two-hybrid assays. Yeast cells transformed with the indicated plasmids were grown at 30°C on control 2DO (SD/-Leu, -Trp) and high-stringency 4DO (SD/-Leu, -Trp, -Ade, -His) plates.

The direct interactions between SOLO and MCM proteins indicate that the establishment of meiotic cohesion in *Drosophila* is linked to DNA replication. Therefore, we investigated whether the function of SOLO in *Drosophila* depends on the replication process, as this is crucial for its meiosis-specific role. To determine whether SOLO must be loaded before or during replication to function effectively, we generated transgenic *Drosophila* expressing Venus::SOLO under the control of *mat*α*4*-GAL4::VP16, in which the *mat*α*4* promoter drives effector expression after DNA replication in the ovary (*55*). By measuring NDJ frequency on the X chromosome, we found that Venus::SOLO, expressed under the control of *mat*α*4*-GAL4::VP16, failed to rescue *solo* phenotypes, as the *solo* mutation still led to more than 40% NDJ. In contrast, the NDJ phenotype resulting from the *solo* mutation was completely rescued when Venus::SOLO expression was driven by *nanos*-GAL4::VP16 (table S4). We also examined the function of SOLO in recombination under the control of *mat*α*4*-GAL4::VP16. As expected, the map distance between *b* and *pr* was markedly reduced, indicating that the function of SOLO in recombination was severely impaired (table S5). Given that SOLO, when expressed under its native promoter, localizes to centromeres of spermatocytes and oocytes before early prophase I (*19*, *20*), these genetic data, together with the direct interaction between SOLO and the MCM complex, suggest that the SOLO-containing cohesin complex is loaded onto chromatin during DNA replication to establish sister chromatid cohesion in *Drosophila* meiosis.

### Conserved SOLO interactions with the mei-MCM complex

SOLO is essential for meiotic recombination and required for the bias toward recombination between homologous chromosomes rather than with sister chromatids in *Drosophila* female meiosis (*19*). However, the precise role of SOLO in meiotic recombination is still unclear. In our yeast two-hybrid screening, we identified direct interactions between DmSOLO and DmMEI-217, as well as between DmSOLO and DmMEI-218 (Fig. 6, A and B). MEI-217 and MEI-218 are subunits of the mei-MCM complex, which is vital for homologous crossover formation during *Drosophila* meiosis (*33*). Given that the splitting of MCMDC2 into MEI-217 and MEI-218 is predominantly exclusive to Diptera (*33*, *56*) and that SOLO interacts with MEI-217 and MEI-218 in *Drosophila*, we investigated the co-occurrence of SOLO with MEI-217 and MEI-218 across dipteran species. Within the suborder Brachycera, the splitting of MEI-217 and MEI-218, along with the presence of SOLO, is evidently not coincidental in both Schizophora and Aschiza (fig. S8). Moreover, SSEP and ORD are also present in these superfamilies of Brachycera. In the suborder Nematocera, SOLO appears to be present in the species containing MEI-217 and MEI-218, such as *Aedes aegypti* in Culicoidea and *Diamesa lindrothi* in Chironomoidea, whereas in the species lacking MEI-217 and MEI-218, such as *Anopheles gambiae* in Culicoidea, no SOLO was identified on the basis of currently available sequence data and bioinformatics analyses. To further confirm the coexistence and interactions of SOLO with MEI-217 and MEI-218 within the same species, we cloned *Zcmei-217* and *Zcmei-218* (gene ID: 105217642). Given that *Zcmei-217* had not yet been annotated in the National Center for Biotechnology Information database, we characterized its nucleotide (accession number: PZ723669) and protein sequences (fig. S9). Consistent with findings from *D. melanogaster*, ZcMEI-217 and ZcMEI-218 strongly interacted with ZcSOLO (Fig. 6, C to E). Moreover, the predicted secondary structure of ZcMEI-217 closely resembles that of DmMEI-217 (fig. S9), suggesting that MEI-217 and MEI-218 in Diptera function in a manner similar to those observed in *D. melanogaster*.

**Fig. 6.**
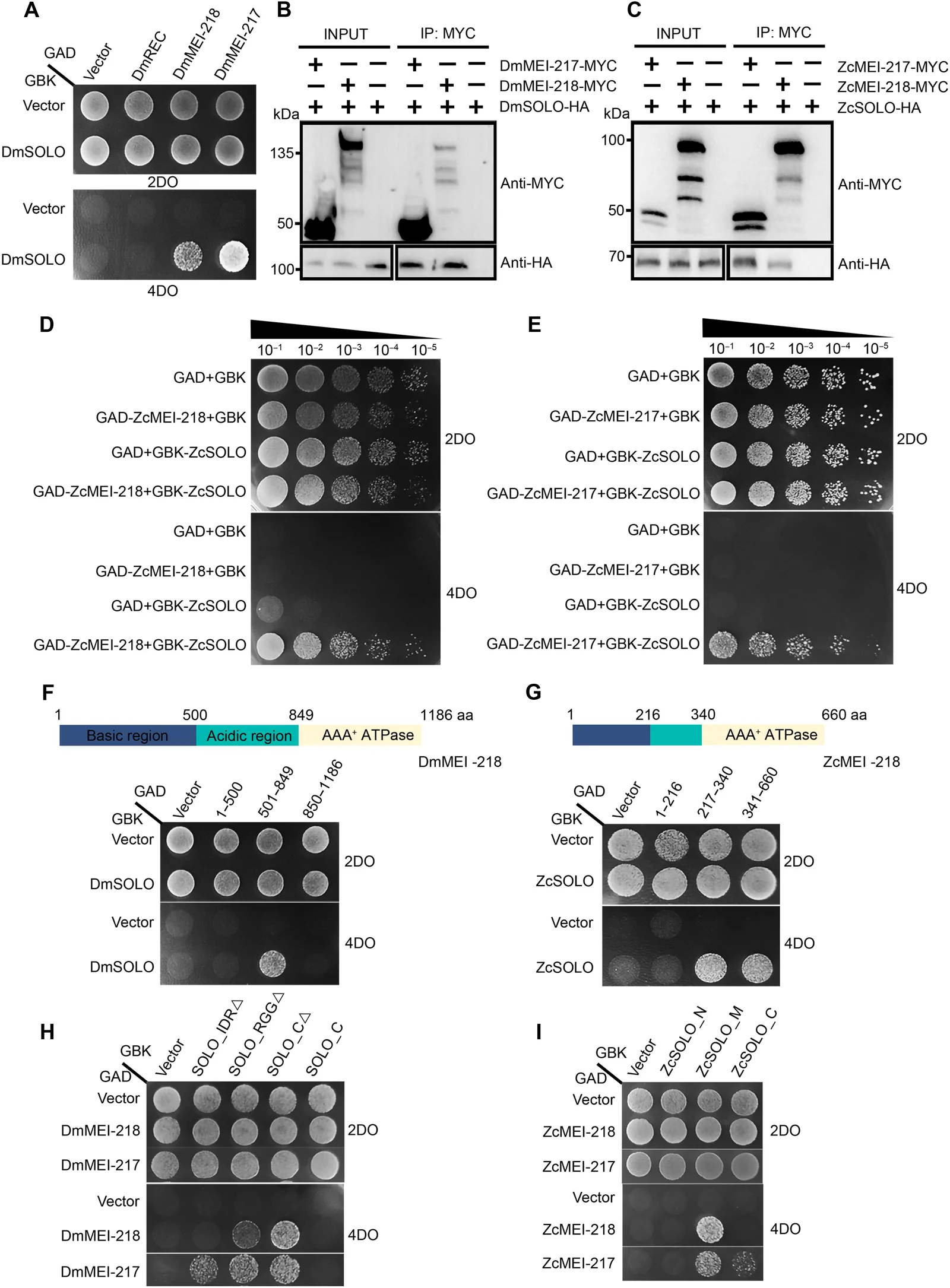
Interaction between SOLO and the mei-MCM Complex. (**A** and **B**) DmSOLO interacts with DmMEI-217 and DmMEI-218 in yeast two-hybrid (A) and Co-IP (B) assays. (**C** to **E**) ZcSOLO interacts with ZcMEI-217 and ZcMEI-218 in Co-IP (C) and yeast two-hybrid (D and E) assays. (**F** and **G**) Yeast two-hybrid assays mapping the domains of MEI-218 that interact with SOLO in *D. melanogaster* and *Z. cucurbitae*. (**H** and **I**) Yeast two-hybrid assays mapping the domains of SOLO that interact with MEI-217 and MEI-218 in *D. melanogaster* and *Z. cucurbitae*.

MEI-218 interacts with MEI-217 through its extended N-terminal region and AAA^+^ adenosine triphosphatase (ATPase) domain; thus, we explored the interaction regions between SOLO and MEI-218. We found that SOLO interacts with a specific region within the extended N-terminal region of MEI-218 in both *D. melanogaster* and *Z. cucurbitae*. In addition, in *Z. cucurbitae*, SOLO interacts with the AAA^+^ ATPase domain of MEI-218 (Fig. 6, F and G). In *D. melanogaster*, MEI-218 and MEI-217 both interact with residues 1 to 662 of SOLO, and deletion of either the intrinsically disordered region or RGG region of SOLO weakens its interaction with MEI-217 (Fig. 6H). In contrast, in *Z. cucurbitae*, MEI-218 and MEI-217 interact exclusively with the middle domain of SOLO (Fig. 6I).

### SOLO function is not affected by mutations in its putative separase cleavage sites

Centromeric REC8 must be protected until metaphase II but cleaved at anaphase II during meiosis to ensure the proper release of sister chromatids. Mutations in the cleavage sites of REC8 result in failed cohesion release and abnormal meiosis (*8*). To investigate whether SOLO can be cleaved by separase, we mutated all potential separase cleavage sites (D/EXXR) in SOLO and generated three transgenic *Drosophila* lines (*solo*^M1^, *solo*^M2^, and *solo*^M3^) to assess their effects on meiosis (fig. S10). Three putative sites in the middle region and all five putative sites in the full length of SOLO were mutated in *solo*^M1^ and *solo*^M2^ mutants, respectively. In *solo*^M3^, all five putative separase cleavage sites and their adjacent residues were simultaneously mutated. Unexpectedly, none of the three kinds of mutations exhibited phenotypes indicative of SOLO dysfunction in the absence of endogenous SOLO. In contrast, the transgenic SOLO with mutated separase cleavage sites nearly fully rescued the *solo* mutant phenotype to wild-type levels (table S6). Therefore, these results suggest that SOLO does not fully replace the REC8 function, distinguishing it from the conventional model of the meiotic cohesin complex.

## DISCUSSION

### A conserved meiotic cohesin complex in Diptera?

Multiple lines of evidence now demonstrate that SOLO fulfills most meiosis-specific roles of REC8 despite the lack of sequence similarity between these two proteins. SOLO is required for the accurate segregation of homologs and sister chromatids in both female and male meiosis, for the co-orientation of sister centromeres in meiosis I, and for the interhomolog recombination bias (*19*, *20*). Moreover, SOLO exhibits genetic interactions with SMC1 and SUNN, the latter of which is thought to be a meiosis-specific homolog of SCC3/SA (*16*, *20*, *21*). In addition, SOLO physically interacts with SMC1 in vivo and consistently colocalizes with it during *Drosophila* male and female meiosis (*19*, *20*). In this study, using yeast two-hybrid assays, we demonstrate that SOLO directly interacts with SMC1 through its C terminus. Mutant flies lacking the SOLO C terminus or carrying mutations of key residues within this region abolish the SOLO-SMC1 interaction. Furthermore, this interaction is evolutionarily conserved between *D. melanogaster* and *Z. cucurbitae*.

The recently identified SSEP exhibits phenotypes and localization patterns similar to those of SOLO in *Drosophila* male meiosis, including precocious sister centromere separation, disrupted co-orientation of sister centromeres, elevated NDJ, and loss of centromeric SUNN localization (*23*). Our study demonstrates that SSEP strongly interacts with SOLO via its C-terminal region and interacts directly with ORD in a conserved manner. These data suggest that the requirement of SUNN for SSEP localization at centromeres is indirect; that is, mutations in *sunn* disrupt the centromeric localization of SOLO, which in turn disrupts centromeric localization of SSEP. Although the roles of SSEP in females have not been characterized, these findings lead us to predict that it functions similarly to SOLO in female meiosis.

In light of the findings presented above, along with the recent research that cohesive cohesion between sister chromatids is conferred by individual cohesin rings (*57*), we propose a model for the meiotic cohesin complex in *Drosophila* and at least some suborders of Diptera (Fig. 7). SOLO interacts with SMC1 through its C-terminal domain and with the C-terminal region of SSEP. SSEP, in turn, interacts with SMC3 through its N-terminal region, as SMC3 can be coimmunoprecipitated by SSEP (Fig. 1E), and the N-terminal region of SSEP exhibits key amino acid residue conservation and similar sequence percent identity matrix to that of REC8 (fig. S5). Given that SMC1 and SMC3 associate with each other to form the canonical SMC1/SMC3 heterodimer within the cohesin complex, these findings suggest that SMC3, SSEP, SOLO, and SMC1 together form a ring structure. SUNN binds to the central region of SOLO to stabilize the entire complex. The cohesin complexes can be interconnected via self-interaction of the SOLO N-terminal region. Given that SOLO, SUNN, SSEP, SMC1, and SMC3 all localize to centromeres of primary spermatocytes during early prophase I and spermatogonia, but ORD does not at the same stages (*18*, *20*, *21*, *23*), we propose that ORD functions as a close partner of the ring structure but rather exerts its role through interaction with SSEP. In conclusion, our results suggest that a noncanonical meiotic cohesin complex exists as a singular entity in Diptera. This configuration differs from the canonical meiotic cohesin complex found in other eukaryotes, highlighting a meiotic cohesion machinery adaptation specific to Diptera.

**Fig. 7.**
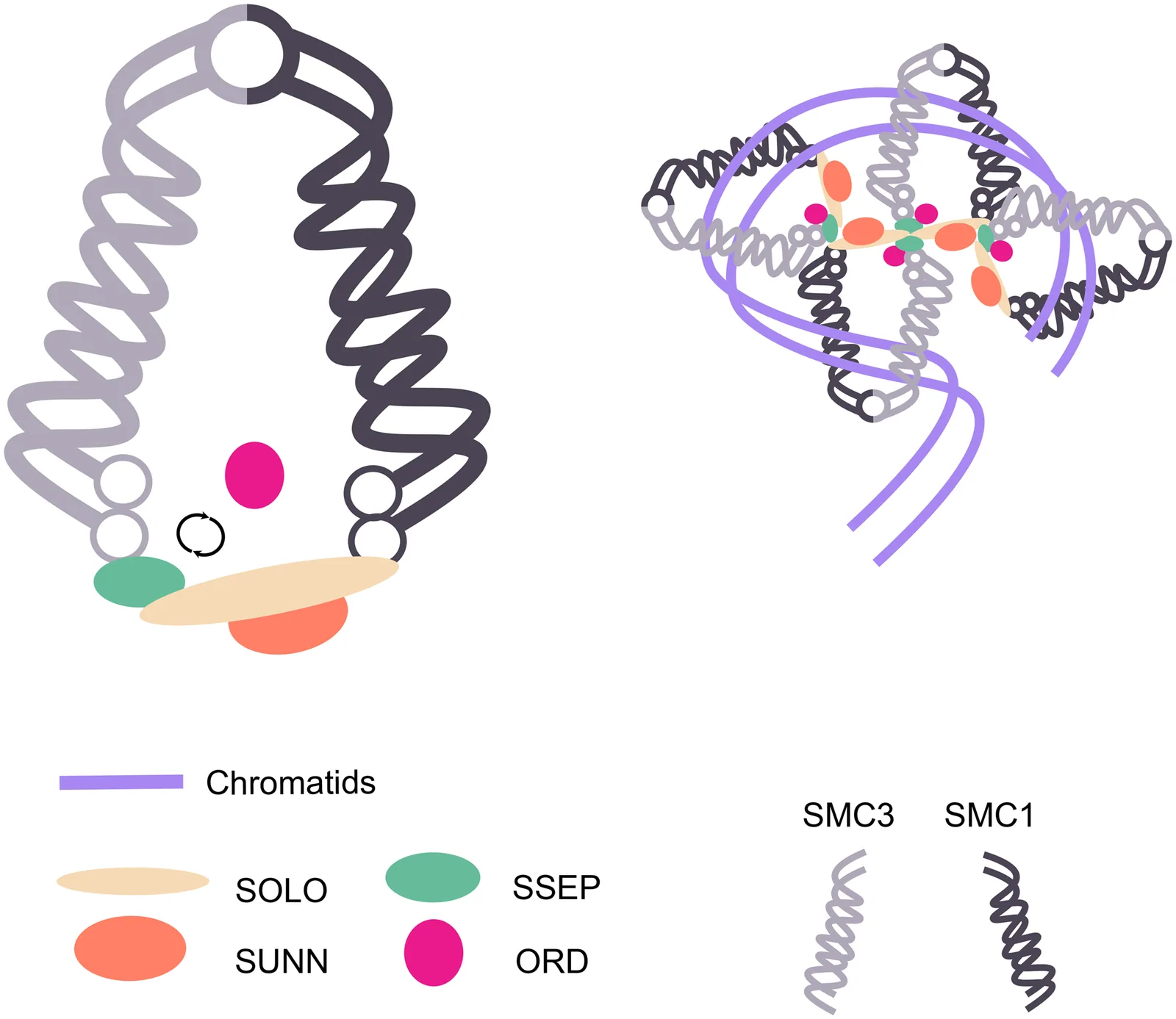
Putative model of the meiotic cohesin complex in Diptera. The N terminus, middle region, and C terminus of SOLO interact with itself, SUNN, and SMC1, respectively. SSEP binds to SOLO and is proposed to interact with SMC3, while ORD associates with the core of the cohesin complex through SSEP. The complex may form multiple ring connections via self-interactions of SOLO and SSEP.

Although we favor the model proposed above, we cannot rule out other possibilities, as two questions still need to be resolved: (i) What is the cleavage target of separase? (ii) Does SSEP or an unidentified protein physically interact with SMC3? Regarding the first question, our data show that mutations at all predicted separase cleavage sites in SOLO do not affect meiotic cohesion or meiosis in *Drosophila*. Mutations of the separase cleavage site in SSEP show only a slight impact on chromosome segregation (*23*). However, both securin and separase are required for *Drosophila* meiosis (*41*, *42*), Moreover, mutations in *mei-S332* cause premature loss of SOLO and SMC1 from centromeres at metaphase I (*20*). Given that separase cleavage sites are typically conserved in α-kleisin proteins, as demonstrated by cleavage experiments with UNO in *Drosophila* (*46*), SCC1 in budding yeast (*58*), and REC8 in fission yeast (*10*), these results raise several possibilities: (i) The separase cleavage sites of SOLO and SSEP are mutually redundant, (2) ORD is the cleavage target of separase, or (3) other unidentified cleavage targets within the cohesin complex may exist, although this possibility seems unlikely. Future experiments, including in vitro cleavage assays and identification of separase interactors, will be necessary to distinguish among these possibilities.

With regard to the second question, SOLO does not appear to be the subunit that interacts with SMC3. First, an interaction between SOLO and SMC3 was not detected (Fig. 1A). Second, the N terminus of SOLO does not interact with SMC3, and its deletion does not disrupt SOLO function in meiosis, likely because the N-terminal sequence of SOLO is not conserved with that of REC8. In yeast, α-kleisin interacts with SMC3 via its N-terminal domain and with SMC1 via its C-terminal domain, forming a ring structure that maintains sister chromatid cohesion (*7*). By contrast, SSEP appears to be the subunit that interacts with SMC3, as its N terminus shows sequence identity and conservation comparable to those of the REC8 N terminus in model organisms (fig. S5). However, we failed to detect a direct interaction between SMC3 and SSEP, SOLO, or ORD. Notably, although SMC3 was expected to interact directly with C(2)M, this interaction was not detectable in our yeast two-hybrid assays (Fig. 1B), even though C(2)M was coimmunoprecipitated with SMC3 from *D. melanogaster* ovary extracts (*15*). To our knowledge, the direct interactions between SMC3 and α-kleisins have not been identified using yeast two-hybrid assays. This failure is likely due to limitations of the yeast two-hybrid system in accurately representing physiological interactions in vivo. SMC3 can be coimmunoprecipitated by SSEP using S2R^+^ cells in the presence of SOLO but not in its absence. This result not only supports the existence of a cohesin complex containing SOLO, SMC1, SMC3, and SSEP but also indicates the limitations of using S2R^+^ cells, which are mitotic in origin, lack meiosis-specific components, and may produce false-negative results. In this study, the cross-validation strategy combining yeast two-hybrid and Co-IP assays effectively minimizes possible false-negative and false-positive results, thereby enhancing the reliability of our findings.

### SOLO, MCM complex, and cohesion establishment

Concomitant with DNA replication in the S phase, cohesin complexes tether sister chromatids together to establish cohesion, likely through different, nonmutually exclusive mechanisms (*57*). In eukaryotes, DNA replication begins with the assembly of pre-RCs, each containing two MCM2-MCM7 complexes (*27*, *28*). Our proteomic and interaction analyses in *D. melanogaster* show that SOLO directly interacts with the MCM complex through its MCM4 and MCM6 subunits. Further supporting this model, MMS22L, a factor associated with the replication fork via CTF4 that links sister chromatid cohesion establishment with DNA replication (*52*, *54*, *59*, *60*), is also a SOLO-interacting protein. Postreplicative ectopic expression of SOLO fails to rescue the high-frequency NDJ phenotype and recombination defects in *solo* mutants. Moreover, SOLO, when expressed under its native promoter, is localized to centromeres in spermatogonia and primary spermatocytes at early prophase I during male meiosis (*20*) and centromeres in oogonia in region 1 and pro-oocytes and oocytes in region 2a of germaria (at zygotene and pachytene) during female meiosis (*19*). Collectively, these data provide substantial evidence that cohesion establishment during *Drosophila* meiosis requires SOLO and is coupled to DNA replication. Physical interactions between cohesin components and pre-RCs have also been observed in mammals, such as the interaction of SMC1 with MCM7 and MCM6 (*30*, *61*, *62*). These findings suggest that the requirement of pre-RCs for both mitotic and meiotic cohesion establishment is likely evolutionally conserved.

It has been proposed that more than one type of cohesin complex exists during meiosis in *D. melanogaster* and *C. elegans*, with each performing distinct functions (*63*, *64*). Furthermore, quantitative studies show that only a small fraction of cohesin complexes reside at centromeres, whereas the majority are associated with chromosome arms (*64*). These observations suggest that the loading or establishment mechanisms of centromeric cohesin complexes may differ from those of arm cohesin complexes. This notion is supported by the findings in *Drosophila* oocytes, where chromatin-associated cohesin turns over extensively and forms new cohesive linkages during meiotic prophase, while the turnover of centromeric cohesin is less extensive (*65*). In addition, in *Mcm5^A7^* mutants, SMC1 accumulation at the centromeres is impaired, whereas cohesin levels on chromosome arms remain largely unaffected (*66*). Therefore, we propose that in *Drosophila*, cohesin loading at centromeres depends on pre-RCs, but loading onto chromosome arms does not. This distinction likely reflects the unique requirement of centromeric cohesin in providing “genuinely cohesive” sister chromatid cohesion.

### Cohesin, mei-MCM complex, and homolog bias

Both SOLO and ORD promote homologous crossovers by suppressing sister chromatid exchange (SCE) in *Drosophila* meiosis, as *solo* and *ord* mutants exhibit a marked increase in SCE in ring-X/rod-X recovery assays (*19*, *32*). This can be explained by the interaction of ORD with SSEP, which in turn interacts with SOLO. A similar role for cohesin in promoting homolog bias has been observed in yeast, where the cohesin components REC8 and SMC3 are required for homolog bias (*11*). This role of cohesin is noteworthy, as sister chromatid cohesion intrinsically favors SCE, yet its disruption paradoxically increases SCE while reducing interhomolog crossovers. Many studies suggest that the axial element (AE) of the SC impedes SCE. The meiosis-specific AE proteins RED1 and HOP1, which are required for homolog bias, physically interact with the meiotic cohesin subunit REC8 (*67*–*69*), which itself is an AE component essential for homolog bias (*11*). Both SOLO and ORD are also AE components in *Drosophila*, suggesting that their contribution to homolog bias may result from their AE roles. However, C(2)M, another AE component in *Drosophila*, is dispensable for sister chromatid cohesion and does not suppress SCE when deleted (*14*). These findings imply that the role of meiotic cohesin in promoting homolog bias through SCE suppression may be coupled to its cohesive function, rather than to its structural role within the AE.

In *Drosophila*, the mei-MCM complex containing MEI-217 and MEI-218 is essential for generating most meiotic crossovers (*5*, *33*, *56*), which arise from homolog bias. Mutations in *mei-218* reduce crossovers and alter their distribution (*38*). In *C. elegans* and *S. cerevisiae*, loss of MSH4 and MSH5, which are functionally substituted by MEI-217 and MEI-218 in Diptera, causes reduced crossovers, altered crossover patterning, and a shift from crossover to noncrossover double-strand break repair (*70*–*73*). These defects parallel to those observed in *solo* mutants: reduced crossovers, altered crossover patterning, and a shift toward noncrossover repair (*19*). Our finding that SOLO directly interacts with MEI-217 and MEI-218 suggests a mechanistic link in which the interaction between the cohesin complex and the recombination machinery is central to its role in homolog bias. This is consistent with the proposed role of MSH4-MSH5 as a sliding clamp that stabilizes Holliday junctions and facilitates homolog bias (*74*). Therefore, the direct interaction of SOLO with MEI-217 and MEI-218 reveals a mechanism through which direct cross-talk between cohesin and the recombination machinery contributes to meiotic homolog bias. DMC1, a meiosis-specific recombinase that directs homology search and is sufficient for normal meiotic interhomolog recombination (*75*), physically interacts with REC8 in both rat (*76*) and yeast (*68*). Thus, the physical connection between cohesin and the recombination machinery appears to be an evolutionarily conserved strategy for ensuring proper homolog bias during meiosis.

Together, current evidence suggests that meiotic homolog bias requires coordination among at least three key groups of factors: the meiotic cohesin, noncohesin AE components, and the recombination machinery. The detailed molecular interplay among these factors remains an intriguing area for future investigation.

### Evolutionary conservation of meiotic cohesion proteins in function and interaction

Our phylogenetic analyses reveal that SOLO, along with its interaction partners SUNN and SSEP, exhibits partial conservation. This conservation is largely restricted to dipteran insects, particularly within families such as Tephritidae and Drosophilidae. In other Diptera species, such as Diopsidae, Glossinidae, and Sarcophagidae, SOLO, SUNN, SSEP, and ORD show similar protein sequences and gene structures. The genomic context of *solo* is intriguing within Diptera. In *Drosophila* and *Zeugodacus*, *solo* is embedded within a large intron of the *vas* gene. We identified a similarly large intron in the *vas* gene of Brachycera species. On the basis of these analyses, we conclude that in species within Brachycera of Diptera, such as Tephritidae and Drosophilidae, SOLO, SUNN, SSEP, and ORD are conserved.

Notably, the presence of SOLO, SSEP, and ORD is strongly correlated with the presence of the mei-MCM complex components. In Diptera, MCMDC2 is split into MEI-218 and MEI-217, with MEI-218 containing an extended N-terminal domain and an AAA^+^ ATPase domain, while MEI-217 contains a conserved MCM N-terminal domain (*33*, *56*). Our interaction studies in *D. melanogaster* and *Z. cucurbitae* reveal that SOLO interacts with both MEI-218 and MEI-217 and that the extended N-terminal domain of MEI-218 is required for its interaction with SOLO. In addition, SOLO, SSEP, and ORD are consistently present with MEI-218 and MEI-217 in the same dipteran species, including Schizophora and Aschiza of Brachycera and Chironomoidea of Nematocera (fig. S8). Therefore, the interaction and constant co-occurrence of SUNN/SOLO/SSEP/ORD and MEI-218/MEI-217 across dipteran species, together with the absence of REC8, suggest that these proteins may be mutually interdependent and may have coevolved a distinctive feature of dipteran meiotic cohesion, contributing to the substantial diversity of reproductive mechanisms observed in insects.

In conclusion, this study demonstrates that SOLO directly interacts with SUNN, SSEP, and SMC1, as well as with various complexes during meiosis. These interactions provide insights into the molecular and biochemical mechanisms underlying the genetic and cellular phenotypes associated with SOLO, SUNN, ORD, and SSEP. Our investigation suggests that the meiotic cohesin complex, through its subunit interactions, orchestrates various protein complexes to execute its diverse functions in meiosis. Furthermore, the interplay between meiotic cohesin and other complexes likely represents a common scenario in Diptera meiosis. Further investigation into the composition of meiotic cohesin complexes in insects will be of utmost importance for elucidating the mechanisms of cohesion in insect meiosis.

## MATERIALS AND METHODS

### Fly stocks and culture methods of *D. melanogaster* and *Z. cucurbitae*

All *Drosophila* flies were maintained at 23°C on standard cornmeal medium. For genetic crosses, parents were removed from the vial on day 10, and progenies were counted between days 13 and 20 (*20*). *Z. cucurbitae* flies were maintained at 26°C, 70% relative humidity, and a light-dark cycle of 14:10 hours. *Z. cucurbitae* larvae were reared on an artificial diet, and adult flies were fed water and a mixture of sugar and brewer’s yeast powder (3:1, w/w) (*77*).

### Generation of *Drosophila* transgenic lines and genetic crosses

Transgenic flies were generated as previously described (*20*). Briefly, the *solo*^M^ open reading frame was inserted downstream of the *Venus* expression cassette in the pPVW plasmid. The recombinant plasmid was then co-injected with the helper plasmid Δ2-3 into *w^1118^* embryos (Center for Excellence in Molecular Cell Science, CAS, Shanghai, China), and transgenic flies were selected on the basis of eye color. The transgenic lines were crossed with GAL4 driver lines. NDJ and recombination frequencies were assessed as previously described (*19*).

### Bioinformatic analyses and structure prediction

Transcript and protein sequences of *vas* and *solo* in Diptera were retrieved from the National Center for Biotechnology Information database (www.ncbi.nlm.nih.gov/). Sequence alignment was performed using MEGA11, and image modifications were executed using GENEDOC. Protein structures were predicted using AlphaFold3 (https://alphafoldserver.com/). Images were generated using ChimeraX.

### Cell culture and transfection

S2R^+^ cells were cultured in Schneider’s medium (no. 21720024, Gibco) supplemented with 10% fetal bovine serum (no. F8318-500 ml, Sigma-Aldrich) and 1% penicillin-streptomycin (no. 15140-122-100 ml, Gibco) in a CO_2_-free incubator at 25°C. The cells were seeded at a density of 5 × 10^6^ cells/ml in 2 ml of complete medium per well and then incubated for 12 hours in a six-well plate. The 100-μl transfection solution consisting of 2 μg of plasmid and 6 μl of FuGENE 6 Transfection Reagent (no. E2691, Promega) in Schneider’s medium was added. Cells were incubated for 48 hours before Co-IP experiments.

### IP–mass spectrometry

Large-scale Co-IPs coupled with mass spectrometry were performed to screen for SOLO-interacting proteins. (i) Ovaries from 200 *Drosophila* females were collected and homogenized in 100 μl of NP-40 lysis buffer (no. FNN0021, Invitrogen) supplemented with 100 mM phenylmethylsulfonyl fluoride (PMSF) and protease inhibitor cocktail (no. 11836153001, Roche). The homogenate was incubated at 4°C for 30 min and then centrifuged at 12,000 rpm for 5 min. The supernatant, as the total protein extract, was collected and precleared by incubation with 4 μl of normal mouse serum (no. NS03L, Sigma-Aldrich) for 1 hour at 4°C. (ii) In a 1.5-ml microcentrifuge tube, 50 μl of protein G agarose (no. 11243233001, Roche) was washed three times with 400 μl of IP solution (NP-40 lysis buffer containing 100 mM PMSF, 1 M dithiothreitol, and 50% glycerol). Each wash was followed by centrifugation at 3000 rpm for 2 min at 4°C, and the supernatant was discarded. (iii) The precleared lysate was then transferred to the tube containing washed protein G agarose and incubated for 3 hours at 4°C. The mixture was subsequently divided into two aliquots. (iv) Ten microliters of either normal mouse serum or monoclonal anti-FLAG antibody (no. MAB3118, Sigma-Aldrich) was added to each aliquot, and the samples were incubated for 5 hours at 4°C. (v) An additional 200 μl of protein G agarose was added to each aliquot and incubated overnight at 4°C. The agarose was collected by centrifugation at 3000 rpm for 2 min at 4°C, and the supernatant was removed. (vi) The agarose was gently washed five times with 400 μl of wash buffer, each time followed by centrifugation at 3000 rpm for 2 min at 4°C. (vii) After the final wash, the supernatant was discarded, and the agarose was resuspended in 25 μl of 2× SDS–polyacrylamide gel electrophoresis loading buffer. The samples were denatured at 95°C for 5 min and stored at −80°C until use. (viii) After validation by Western blotting and Coomassie staining, gel slices corresponding to the experimental and control samples were excised and sent to BGI Genomics (Beijing, China) for mass spectrometry analysis to identify potential SOLO-interacting proteins.

### Coimmunoprecipitation

SOLO, SUNN, and SMC1 were cloned into pUASt-3HA and pUASt-13MYC vectors using seamless cloning (no. RK21020, ABclonal), respectively. The primer sequences used in this study are available upon request. Proteins were expressed in S2R^+^ cells using the UAS-GAL4 system with cotransfection of pUASt-SOLO-13MYC, pUASt-SUNN-3HA, and pCaSper4-Act5C-GAL4 (provided by W. Zhang, Nanjing Agricultural University, China). Transfected S2R^+^ cells were dissociated using trypsin (no. abs47014938, Absin) and collected by centrifugation at 5000*g* for 5 min at 4°C in 2-ml tubes. The cells were washed with cold phosphate-buffered saline [137 mM NaCl, 2.7 mM KCl, 1.47 mM KH_2_PO_4_, and 6.46 mM Na_2_HPO_4_ (pH 7.4)] and then transferred to 1.5-ml Protein LoBind Tubes (no. 0030108116, Eppendorf). Subsequent procedures were conducted at 4°C, including cell lysis with lysis buffer [50 mM tris-HCl (pH 8.0), 150 mM NaCl, 1% NP-40, and 1 mM PMSF] for 2 hours. After centrifugation at 12,000*g* for 5 min, the supernatant was transferred to 1.5-ml Protein LoBind tubes, followed by incubation with 20 μl of Pierce Anti–c-Myc Agarose (no. 20169, Thermo Fisher Scientific) for 2 hours. The agarose was washed three times with wash buffer [50 mM tris-HCl, 150 mM NaCl, and 1 mM PMSF (pH 7.4)] for 5 min each. One hundred microliters of protein loading buffer (no. AR1112-10, Boster) was added, and then the mixture was heated at 95°C for 5 min to release proteins from the agarose.

### Western blotting

The protein solutions eluted from the IP experiments were separated on 8% SDS–polyacrylamide gel electrophoresis gels. The primary antibodies used in this study were 1:5000 anti-HA (no. HT301-01, TransGen, mouse) and 1:5000 anti-Myc (no. HT101-01, TransGen, mouse). The secondary antibody was 1:10000 horseradish peroxidase–conjugated goat anti-mouse (no. HS201-01, TransGen). Signal detection was performed using an enhanced chemiluminescence kit (no. P0018AS, Beyotime).

### Yeast two-hybrid assay

To explore protein-protein interactions, such as SOLO and SUNN of *D. melanogaster* and SOLO and SMC1 of *Z. cucurbitae*, we used the GAL4-based Matchmaker Yeast Two-Hybrid System 3 (Clontech Laboratories), following the methodology described by Zeng *et al.* with modifications (*78*). Briefly, the specified genes, derived from cDNA libraries of either *D. melanogaster* or *Z. cucurbitae*, were fused to the GAL4 activation domain in the pGADT7 vector or the GAL4 DNA-binding domain in the pGBKT7 vector. These corresponding pairs of constructs were cotransformed into the tester strain AH109, in which the *HIS3* and *ADE2* reporter genes are governed by three unrelated GAL4-responsive UAS elements and the *GAL1* and *GAL2* promoters. Transformants were first cultured on 2DO (SD/-Leu, -Trp) medium and subsequently plated on 3DO (SD/-Leu, -Trp, -His) and 4DO (SD/-Ade, -His, -Leu, -Trp) medium.

### Image processing

All image processing operations, including color and contrast adjustments, were carried out using Adobe Photoshop 2023.
