# Supplementary material for: A noncanonical meiotic cohesin complex associates with specific MCM complexes to promote cohesion establishment and homolog bias
Source: Sci Adv. 2026 Oct 23;12(43):eaej1008. doi: 10.1126/sciadv.aej1008 (PMC13693663; doi:10.1126/sciadv.aej1008)
Supplement: Supplementary file 1 [file sciadv.aej1008_sm.pdf]

Science Advances
AAAS

Supplementary Materials for

A noncanonical meiotic cohesin complex associates with specific MCM complexes to promote cohesion establishment and homolog bias

Lujie Ma *et al.*

Corresponding author: Rihui Yan, ryan1@hainanu.edu.cn

Sci. Adv. **12**, eaej1008 (2026)
DOI: 10.1126/sciadv.aej1008

This PDF file includes:

Figs. S1 to S10
Tables S1 to S6

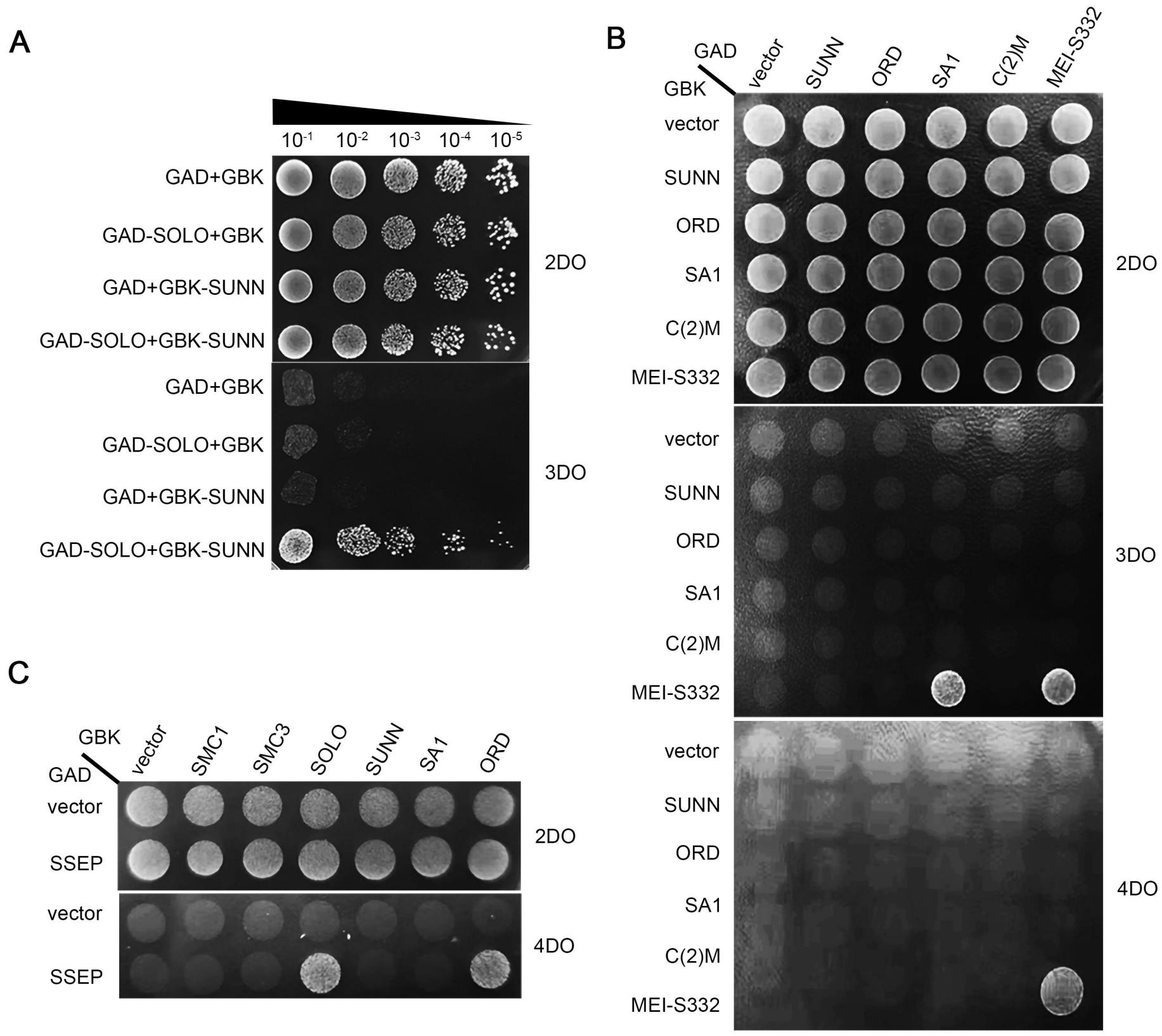

fig. S1. Yeast two-hybrid analyses of interactions between meiotic proteins.
(**A** to **C**) Yeast cells transformed with the indicated plasmids were grown at 30°C on control 2DO (SD/-Leu, -Trp) and high-stringency 3DO (SD/-Leu, -Trp, -His), 4DO (SD/-Leu, -Trp, -Ade, -His) plates.

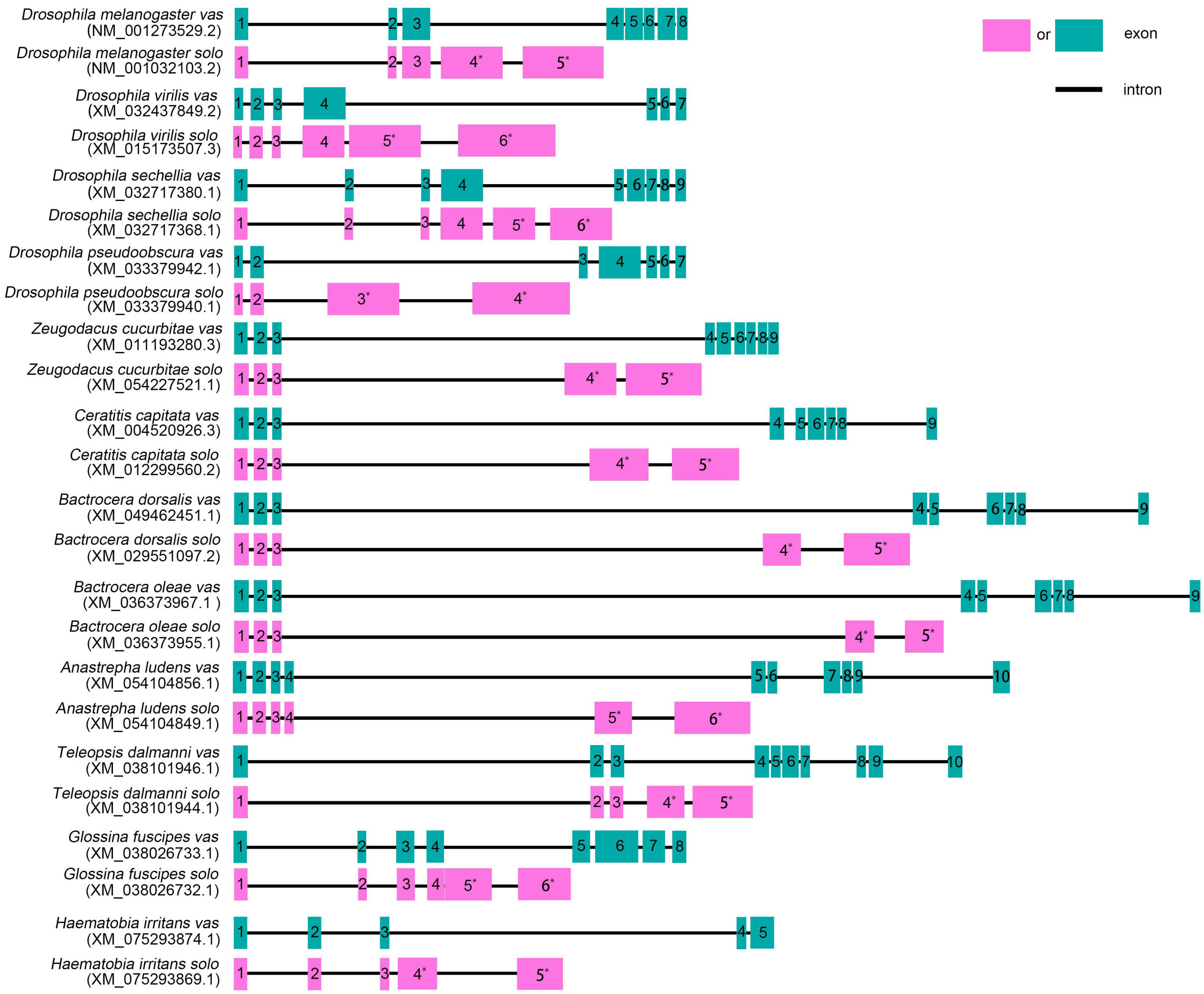

fig. S2. Schematic diagrams of the gene structures of *vas* and *solo* in different dipteran insects. The intron and exon lengths are not scaled to their actual sizes.

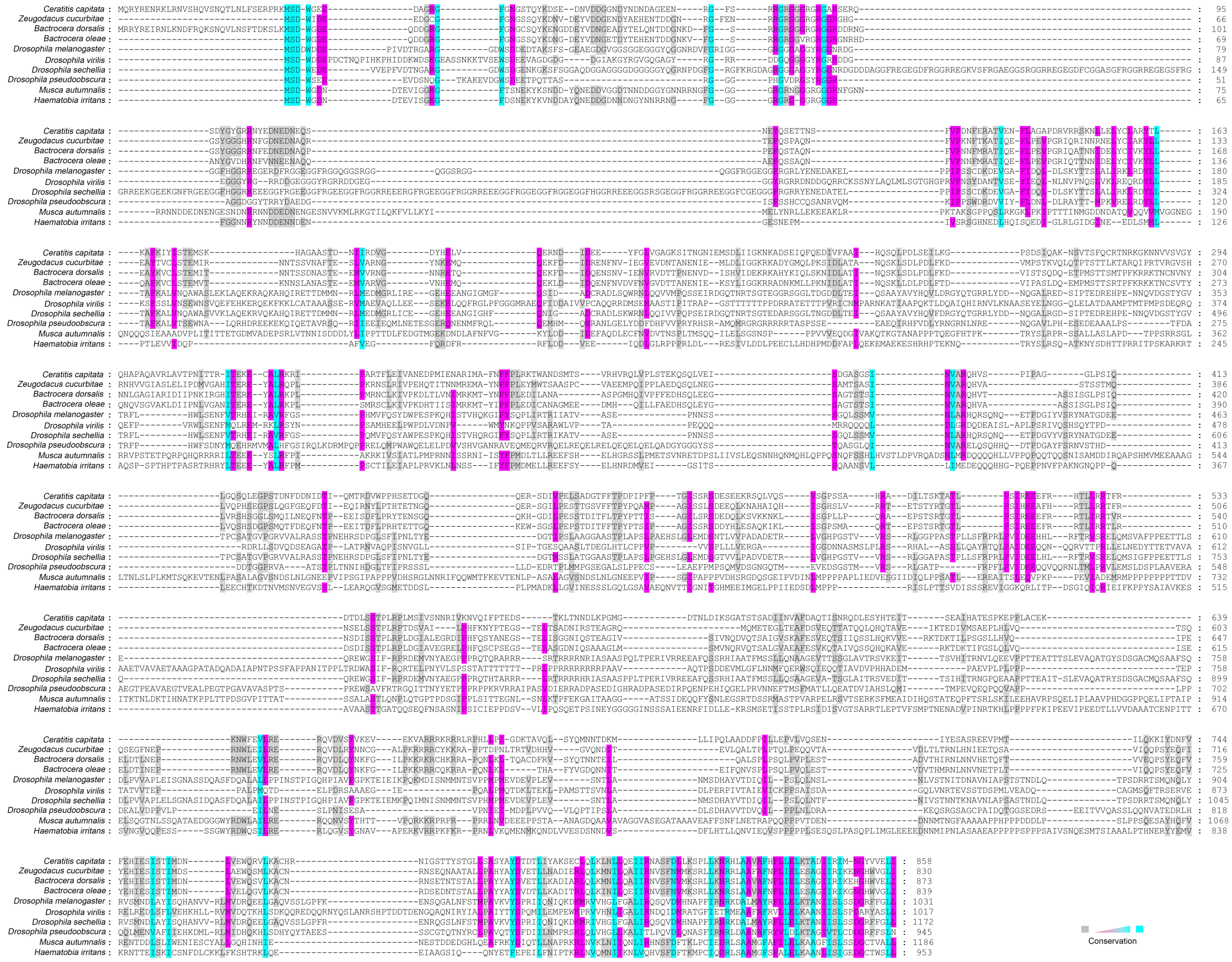

fig. S3. Sequence alignment of SOLO proteins in Tephritidae, Drosophilidae, and Muscidae. Amino acid conservation is indicated by increasing color intensity from white to gray, pink, and blue. The protein sequences used for alignment were obtained from GenBank under the following accession numbers: *C. capitata* (XP_012154950.1), *Z. cucurbitae* (XP_054083496.1), *B. dorsalis* (XP_049318406.1), *B. oleae* (XP_036229847.2), *D. melanogaster* (NP_001027274.2), *D. virilis* (XP_015028993.1), *D. sechellia* (XP_032573259.1), *D. pseudoobscura* (XP_033235830.1), *M. autumnalis* (XP_073823667.1), *H. irritans* (XP_075149984.1).

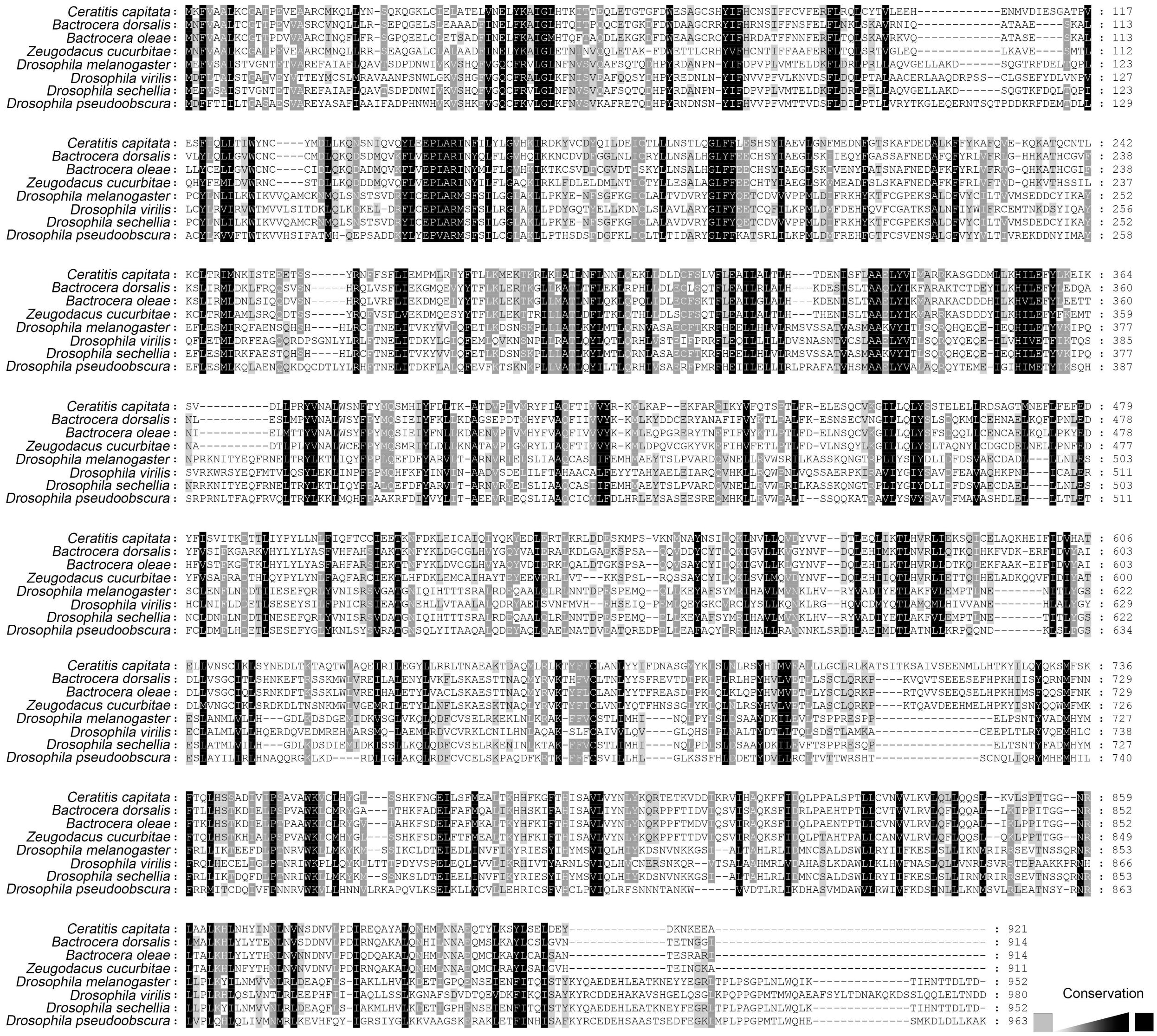

fig. S4. Sequence alignment of SUNN proteins in Tephritidae and Drosophilidae.
Amino acid conservation is indicated by increasing color intensity from white to gray to black. The protein sequences used for alignment were obtained from GenBank under the following accession numbers: *C. capitata* (XP_020713819.1), *B. dorsalis* (XP_011211982.2), *B. oleae* (XP_036232851.2), *Z. cucurbitae* (XP_054088431.1), *D. melanogaster* (NP_729739.3), *D. virilis* (XP_002048619.3), *D. sechellia* (XP_032574381.1), *D. pseudoobscura* (XP_001352778.4).

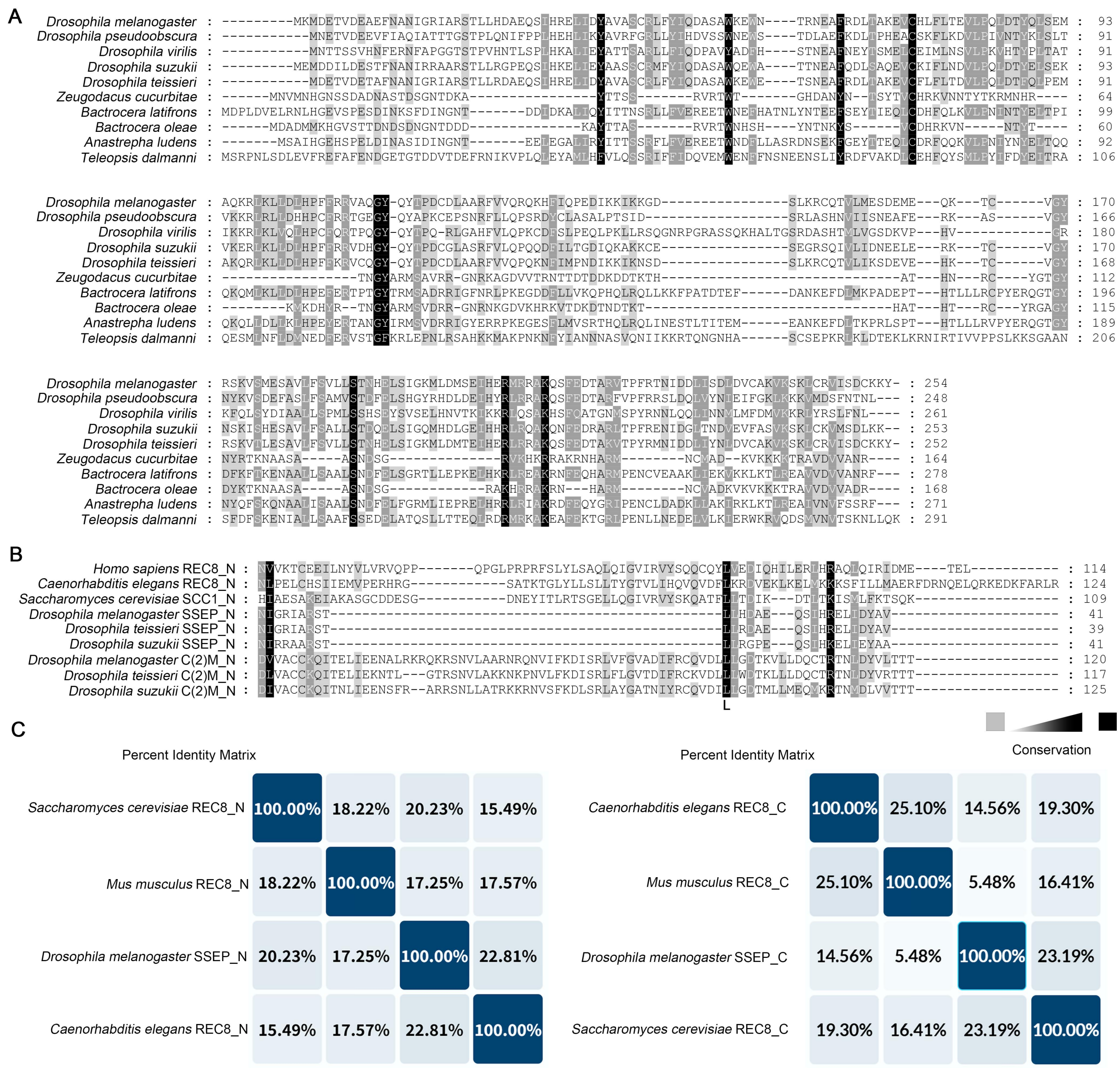

fig. S5. Sequence alignment of SSEP proteins in Acalyptratae.

(**A**) Sequence alignment of SSEP in full length. (**B**) Sequence alignment of SSEP N-terminal region. Amino acid conservation is indicated by increasing color intensity from white to gray to black. The SSEP protein sequences used for alignment were obtained from GenBank under the following accession numbers: *D. melanogaster* (NP_724612.2), *D. pseudoobscura* (XP_002135475.2), *D. virilis* (XP_002047640.1), *D. suzukii* (XP_016928351.2), *D. teissieri* (XP_043662723.1), *Z. cucurbitae* (XP_011186387.1), *B. latifrons* (XP_018803452.1), *B. oleae* (XP_014088358.2), *A. ludens* (XP_053954623.1), *T. dalmanni* (XP_037937564.1), *H. sapiens* REC8 (NP_001041670.1), *C. elegans* REC8 (NP_001255727.1), *S. cerevisiae* SCC1 (NP_010281.1), *D. melanogaster* C(2)M (NP_609788.1), *D. Suzukii* C(2)M (XP_016945876.3), *D. teissieri* C(2)M (XP_043640592.1). (**C**) Conservation analysis of the N- and C-terminal regions of SSEP with those of REC8 using the UniProt online tool (https://www.uniprot.org/align).

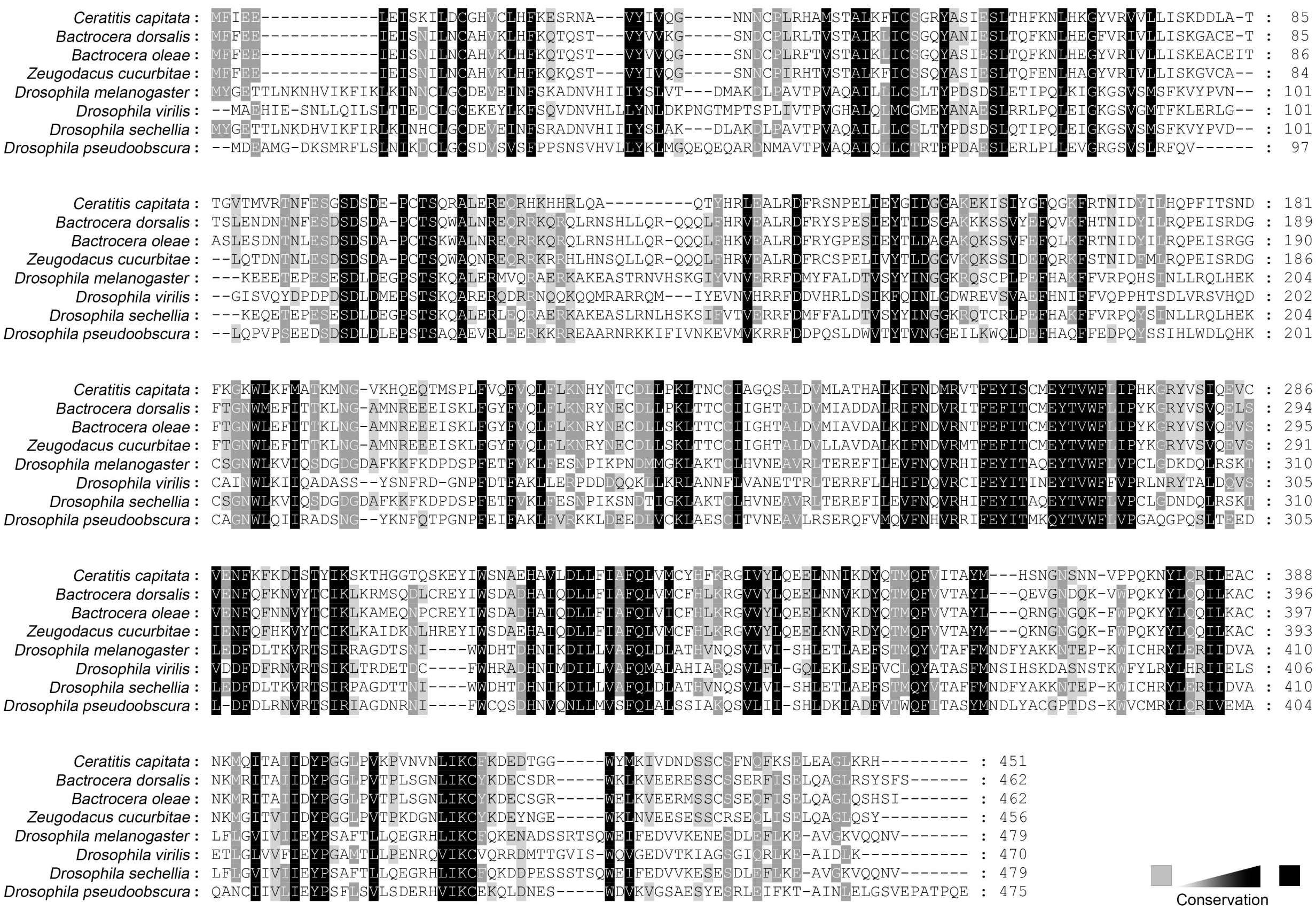

fig. S6. Sequence alignment of ORD proteins in Tephritidae and Drosophilidae.
Amino acid conservation is indicated by increasing color intensity from white to gray to black. The protein sequences used for alignment were obtained from GenBank under the following accession numbers: *C. capitata* (XP_004523724.2), *B. dorsalis* (XP_019847914.2), *B. oleae* (XP_014091646.2), *Z. cucurbitae* (XP_054087160.1), *D. melanogaster* (NP_001286760.1), *D. virilis* (XP_002049053.1), *D. sechellia* (XP_032572478.1), *D. pseudoobscura* (XP_001360390.2).

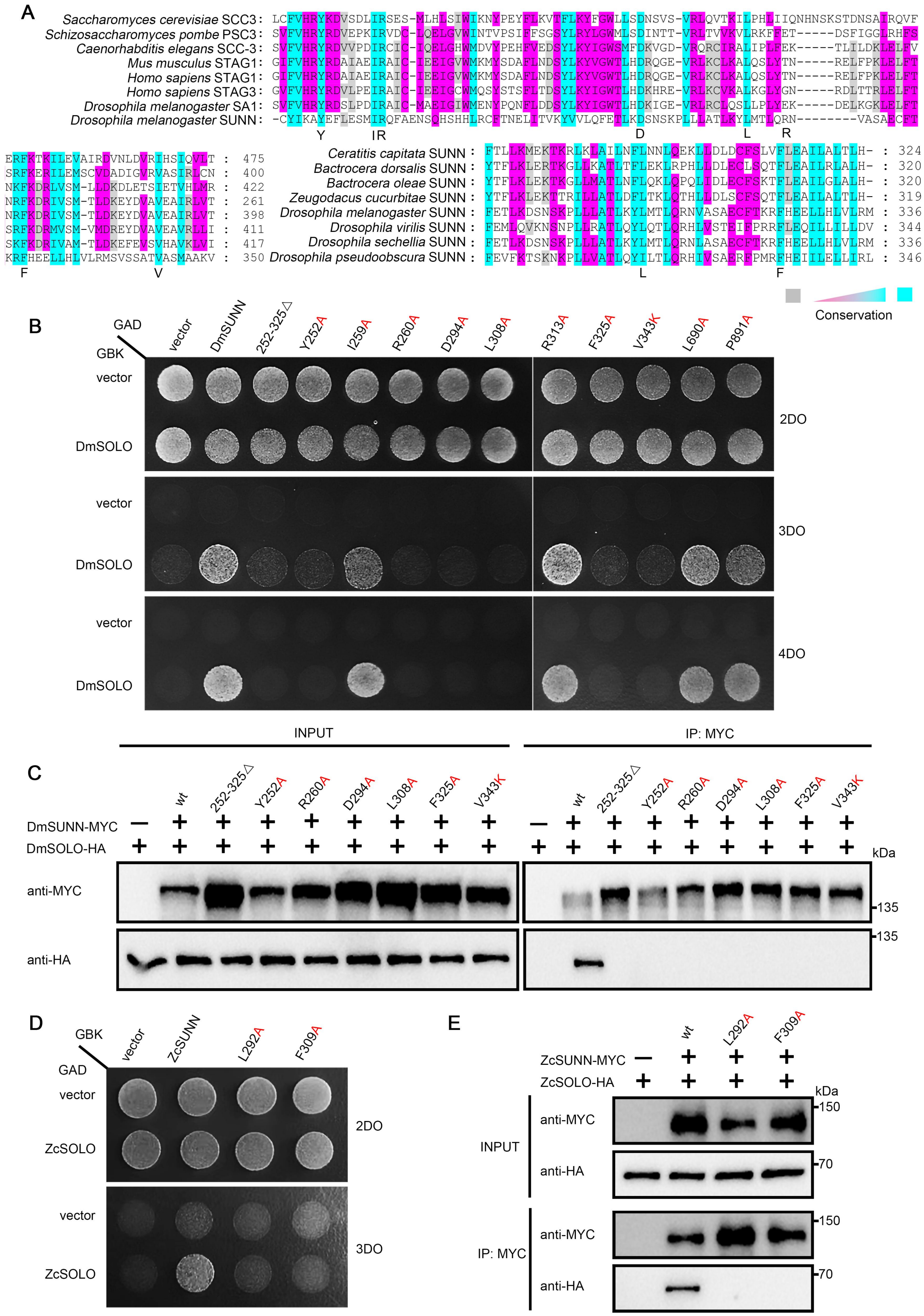

fig. S7. Identification of conserved amino acid residues required for the SUNN-SOLO interaction.

(**A**) Sequence alignment of SUNN and SA from model organisms *S. cerevisiae*, *S. pombe*, *C. elegans*, *M. musculus* and *H. sapiens*. Amino acid conservation is indicated by increasing color intensity from white to gray, pink, and blue. The protein sequences used for alignment were obtained from GenBank under the following accession numbers: *S. cerevisiae* (NP_012238.1), *S. pombe* (NP_001343016.1), *C. elegans* (NP_506193.1), *M. musculus* (NP_001344193.1), *H. sapiens* STAG1 (NP_005853.2), *H. sapiens* STAG3 (NP_001269646.1), *D. melanogaster* SA1 (NP_477268.2), *D. melanogaster* SUNN (NP_729739.3), *C. capitata* (XP_020713819.1), *B. dorsalis* (XP_011211982.2), *B. oleae* (XP_036232851.2), *Z. cucurbitae* (XP_054088431.1), *D. virilis* (XP_002048619.3), *D. sechellia* (XP_032574381.1), *D. pseudoobscura* (XP_001352778.4). (**B** to **E**) Yeast two-hybrid (**B** and **D**) and Co-IP (**C** and **E**) assays assessing the effects of mutations in conserved residues of *D. melanogaster* and *Z. cucurbitae* SUNN on its interaction with SOLO.

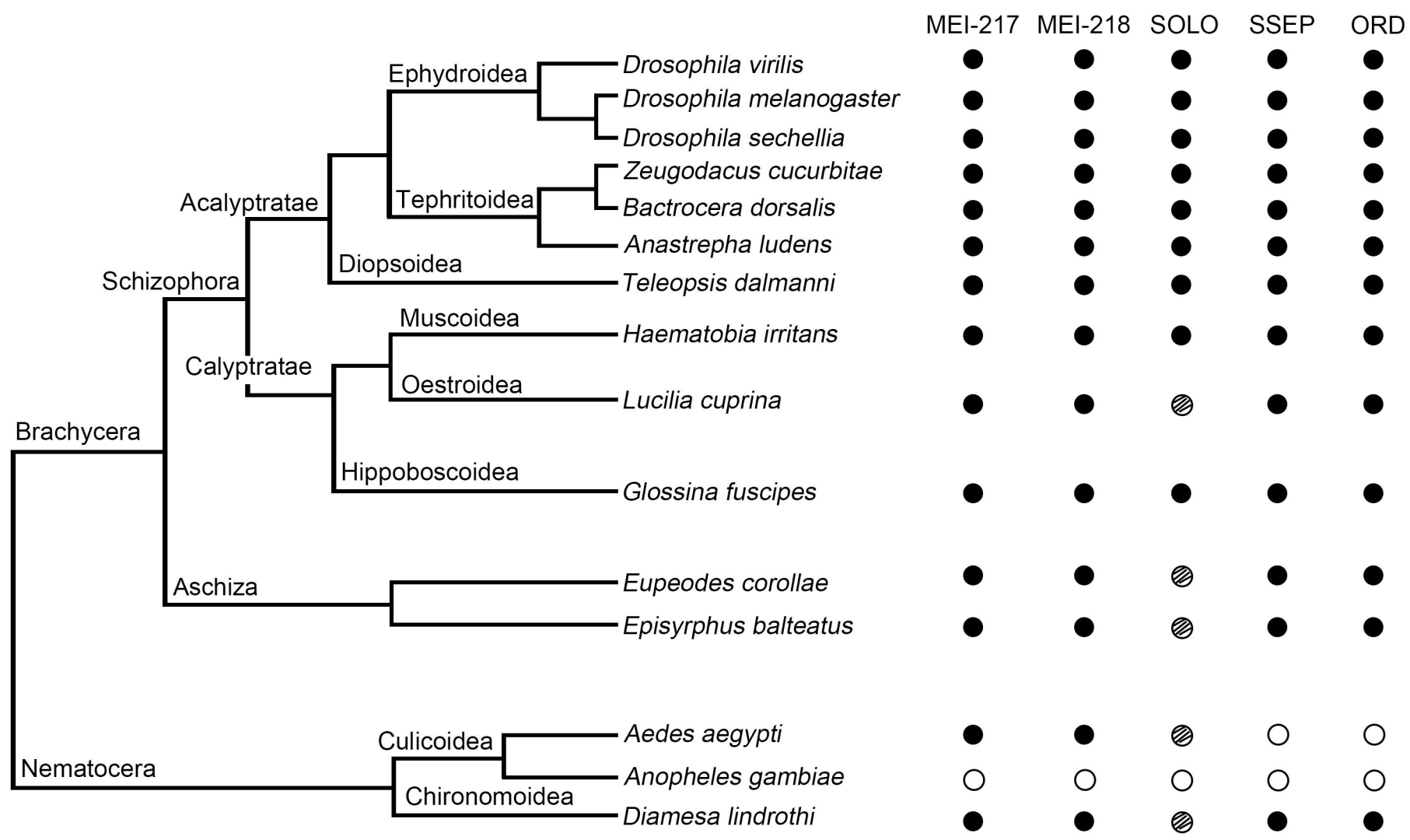

fig. S8. Occurrence of MEI-217, MEI-218, SOLO, SSEP, and ORD in Diptera.
The dendrogram on the left was constructed as described in Hartmann et al. (2019), showing the relationships among dipteran taxa (*56*). On the right, filled circles indicate confident presence of the protein in the corresponding species; open circles indicate that the protein has not yet been found in GenBank; shaded circles indicate that, although not found in GenBank, the presence of SOLO is inferred from the large intron structure of the *vas* gene.

>*Zeugodacus cucurbitae mei-217* CDS

ATGTTCAATGTAGGGGCTATATTCATTGGAAGGGATAACGATTCTTCGCAAATATCGCAGTCA
TTCCGAGATCAAAAACTCCTTGAAGTATTTATGCGACGCGGTCAAATGATCAAAATTATTGAA
GGCGCAACCGAACAATTCATAGAAAGATATCGTAATAGCAAGAAGCCGGAAGACGCATTAGG
TGCTTTATTTGTACCGCTCCATTTCGAGTACAGCGATTTAATTGAGACTCATGCAATTGAATTG
CTGCATCAGGTTATCGAAGAACCATTACAATTTCTGAACGCTGCTAAATATTGCGTGTATGGAA
TTGTTCGTGACCAAATTAAATTGTCTCGAAACACAGAAGAAAGTAACGCACCAAGGGCCATCG
ACATTGACCAGCTACATTTACAGCTGCGATTCAACGGCTTACCACTACAGGAAGATTTGCACTT
TGCAAACTTCAAAAACGGCTGGCCACAAGGGCTGTCGTGGACAATTGGAATTTTATCAGCGATA
TCTGAACCTCAGTTAGAAATTTTACAGTCGACGTGGTTTTGTTCAACCGGTTGCCAGCGCAACA
AAATTAAATCAGACTCAGTAGACGCTCCTATATGCCACAGTTGTGGTCATCACATGAATGAATAT
GCAAAATTGCGTGTGACTGAGAAATTTCACTTGCTGCGCTTACTTCCCCCCTCTTGTATTGAGAG
TCCCAGAGTTGAGAATAAAATTTTTCGTGCACTAACTGTGCATGTCAAAGAACACGTCCACGATT
GCGAGCTTCGTCTTGGTAACAGCTATCTCATAATTGGCTACTACGATTATACGCGCGCTGGTCAA
ATTTTTCAAGCATGGAGCGTAATCACGCATTAG

>*Zeugodacus cucurbitae* MEI-217 protein

MFNVGAIFIGRDNDSSQISQSFRDQKLLEVFMRRGQMIKIIEGATEQFIERYRNSKKPEDALGALF
VPLHFEYSDLIETHAIELLHQVIEEPLQFLNAAKYCVYGIVRDQIKLSRNTEESNAPRAIDIDQLHLQ
LRFNGLPLQEDLHFANFKNGWPQGLSWTIGILSAISEPQLEILQSTWFCSTGCQRNKIKSDSVDA
PICHSCGHHMNEYAKLRVTEKFHLLRLLPPSCIESPRVENKIFRALTVHVKEHVHDCELRLGNSY
LIIGYYDYTRAGQIFQAWSVITH

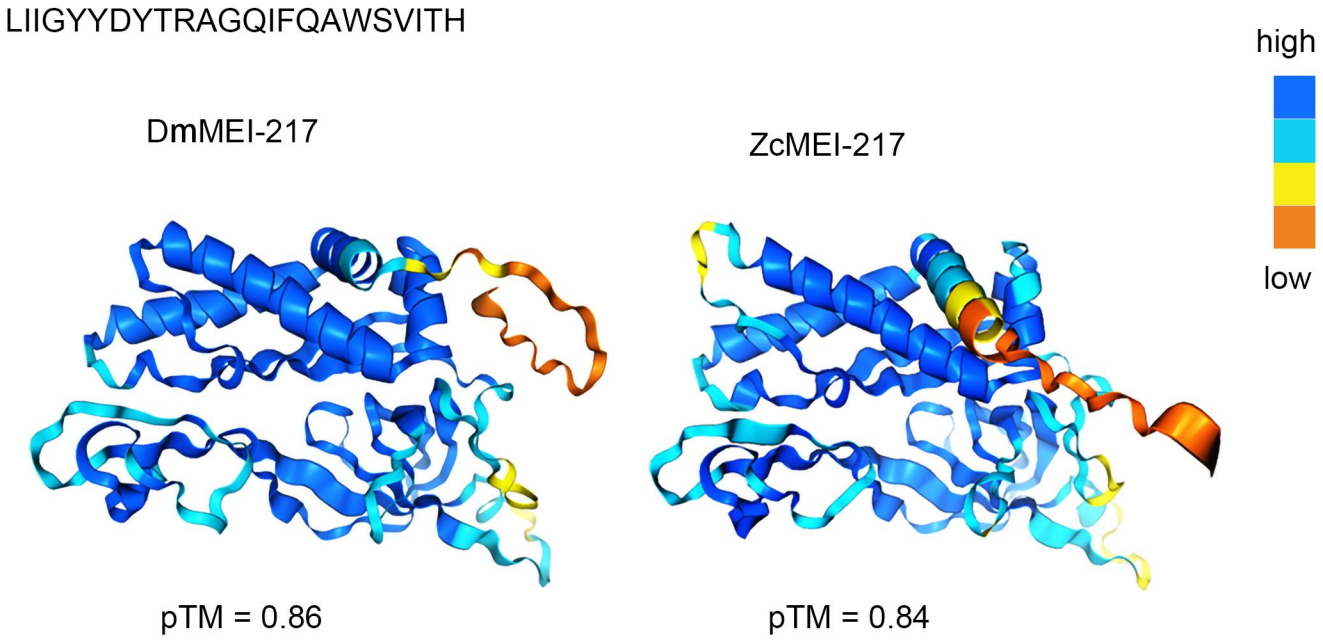

fig. S9. Nucleotide and protein sequences of ZcMEI-217.
The MEI-217 structures in *D. melanogaster* and *Z. cucurbitae* were predicted by AlphaFold3. Structural prediction confidence is indicated by increasing color intensity from orange to blue.

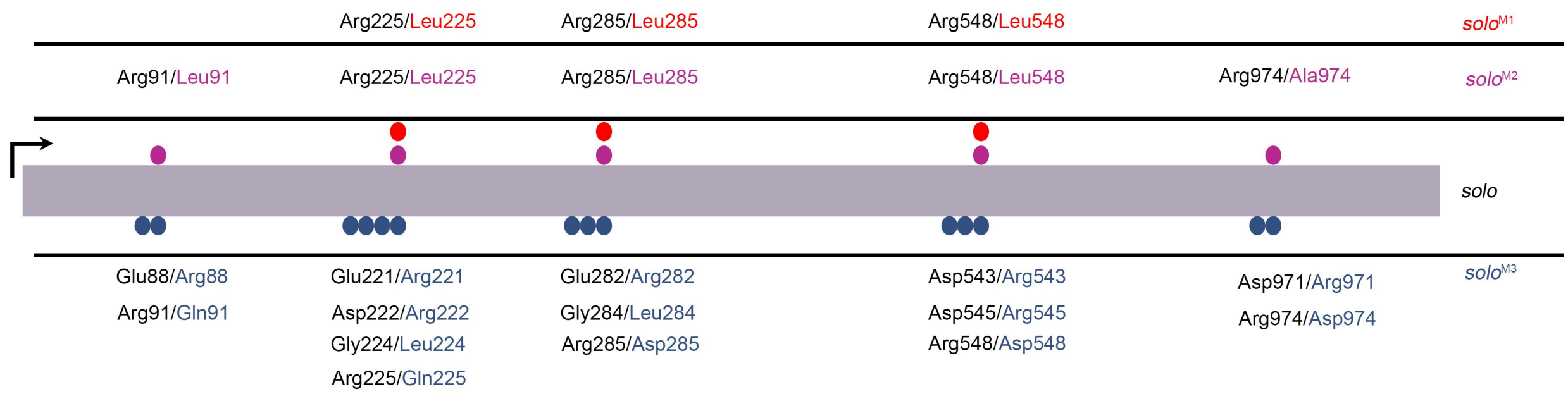

fig. S10. Molecular characterization of putative SOLO separase cleavage site mutants. Schematic representation of three transgenic mutants with amino acid substitutions at putative separase cleavage sites of SOLO, used to assess their effects on SOLO function. M1 mutant: simultaneous mutation of three putative separase cleavage sites (D/EXXR) in the middle region of SOLO. M2 mutant: simultaneous mutation of all five putative separase cleavage sites of SOLO. M3 mutant: simultaneous mutation of all five putative separase cleavage sites and adjacent residues of SOLO as described previously (*58*).

table S1. Sex chromosome NDJ in transgenic flies

Male Genotype	N*	%NDJ (n†)
solo/+, UASp-Venus::SOLO$^{N\Delta}$ or *nanos*-GAL4::VP16/+	368	0.5 (2)
solo/Df, UASp-Venus::SOLO$^{N\Delta}$/*nanos*-GAL4::VP16	483	3.93 (19)
solo/Df, UASp-Venus::SOLO$^{RGG\Delta}$/*nanos*-GAL4::VP16	483	42.2 (204)
solo/Df, UASp-Venus::SOLO$^{N\Delta}$ or *nanos*-GAL4::VP16/+	328	53.9 (117)

w/*B^{s}Yy^{+}* males with the indicated chromosome genotypes were crossed singly to 2-3 *y w* females. *Df* = *Df(2L)A267*, in which the *solo* locus is completely deleted. * total number of progeny scored. † abnormal (disjunctional) progeny.

table S2. Sex chromosome NDJ in transgenic flies

Male Genotype	N*	%NDJ (n†)
solo/+, UASp-Venus::ZcSOLO or *nanos*-GAL4::VP16/+	555	0.5 (3)
solo/Df, UASp-Venus::SOLO_2/*nanos*-GAL4::VP16	334	36.5 (122)
solo/Df, UASp-Venus::ZcSOLO/*nanos*-GAL4::VP16	241	58.0 (125)
solo/Df, UASp-Venus::ZcSOLO or *nanos*-GAL4::VP16/+	200	56.6 (113)

w/*B^{s}Yy^{+}* males with the indicated chromosome genotypes were crossed singly to 2-3 *y w* females. *Df* = *Df(2L)A267*, in which the *solo* locus is completely deleted. * total number of progeny scored. † abnormal (disjunctional) progeny.

table S3. Summary of SOLO-interacting proteins identified by FLAG purification

	Repeat 1#		Repeat 2#		Repeat 3#		Repeat 4#		
Name	Pep*	%Co†	Pep*	%Co†	Pep*	%Co†	Pep*	%Co†	Description
IP: SOLO	13	22	13	20	22	30	22	30	Cohesin subunit
MCM2	7	16	3	4	14	25			Cohesin loading/ Establishme nt
MCM3	16	29			12	28	10	21	
MCM4	11	28	1	2	14	29			
MCM5	9	26			10	27			
MCM6	4	7			8	16			
MCM7	10	26	2	4			7	25	
MMS22L	1	1	1	1	1	1	1	0	
Nipped-B	2	3			5	6			
MEI-9	2	4			2	4			DNA repair
MEI-41	1	3			2	1			
SMC1			1	3	1	1	2	6	Cohesin subunit
SMC3			3	2	2	10	1	3	
SUNN							1	3	

* Peptides, † %Coverage.

table S4. Sex chromosome NDJ in transgenic flies

Female Genotype	N*	%NDJ (n†)
w/+, solo/Df, UASp-Venus::SOLO; *nanos*-GAL4::VP16	713	0% (0)
w/w, solo/Df, UASp-Venus::SOLO; *matα4*-GAL4::VP16	357	62.7% (163)

w/w famales with the indicated chromosome genotypes were crossed singly to 2-3 $Y^S X Y^L$, *In(1)En, yB* males. *Df* = *Df(2L)A267*, in which the *solo* locus is completely deleted. * total number of progeny scored. † abnormal (disjunctional) progeny.

table S5. 2nd chromosome recombination in transgenic flies

Female Genotype	Map Distance		
	b cn	*cn bw*	N*
solo/b vas⁷ pr, UASp-Venus::SOLO; *matα4*-GAL4::VP16	5.0 cM	5.0 cM	80
solo/+, UASp-Venus::SOLO; *matα4*-GAL4::VP16	ND	36.8 cM	190

* total number of progeny scored.

table S6. Sex chromosome NDJ in transgenic flies

Male Genotype	N*	%NDJ (n†)
solo/+, UASp-Venus::SOLO^{M1} or *nanos*-GAL4::VP16/+	1845	0.33 (6)
solo/Df, UASp-Venus::SOLO^{M1} or *nanos*-GAL4::VP16/+	349	47.6 (166)
solo/Df, UASp-Venus::SOLO^{M1}/*nanos*-GAL4::VP16	857	0.30 (3)
solo/Df, UASp-Venus::SOLO^{M2} or *nanos*-GAL4::VP16/+	377	48.5 (183)
solo/Df, UASp-Venus::SOLO^{M2}/*nanos*-GAL4::VP16	993	0.20 (2)
solo/Df, UASp-Venus::SOLO^{M3} or *nanos*-GAL4::VP16/+	1127	46.6 (525)
solo/Df, UASp-Venus::SOLO^{M3}/*nanos*-GAL4::VP16	857	0.46 (4)

w/B^sYy^+ males with the indicated chromosome genotypes were crossed singly to 2-3 *y w* females. *Df* = *Df(2L)A267*, in which the *solo* locus is completely deleted. * total number of progeny scored. † abnormal (disjunctional) progeny.
